# Computer Vision-Based Techniques for Conveyor Belt Condition Monitoring: A Systematic Review

**DOI:** 10.3390/s26082527

**Published:** 2026-04-20

**Authors:** Pablo Rios-Colque, Victor Rios-Colque, Luis Rios-Colque, Pedro A. Robles

**Affiliations:** 1 Ingeniería Mecánica-Electromecánica-Mecatrónica, Facultad Nacional de Ingeniería, Universidad Técnica de Oruro, Oruro, Bolivia; pablo.rios.col@gmail.com (P.R.-C.); victor.r.rios.c@gmail.com (V.R.-C.); 2 Doctorado en Industria Inteligente, Facultad de Ingeniería, Pontificia Universidad Católica de Valparaíso, Valparaíso 2340000, Chile; luis.rios.c@mail.pucv.cl; 3 Escuela de Ingeniería Química, Facultad de Ingeniería, Pontificia Universidad Católica de Valparaíso, Valparaíso 2340000, Chile

**Keywords:** computer vision, conveyor belt, machine learning, artificial intelligence, image processing, condition monitoring, maintenance, smart industry

## Abstract

**Highlights:**

**What are the main findings?**

- The main applications of computer vision-based techniques for conveyor belt monitoring are damage detection, deviation detection, and foreign object detection.
- A transition is observed from traditional image processing methods toward deep learning models, with improvements in precision, speed, and stability.

**What are the implications of the main findings?**

- The advances strengthen condition monitoring, but more representative datasets and models with lower computational complexity are required.
- There are opportunities for multimodal, lighter, and more adaptive solutions that facilitate integration into asset management platforms in mining environments.

**Abstract:**

Conveyor belts are critical equipment in mining operations, where continuous and reliable material transport is essential for production efficiency. This systematic review aims to analyze computer vision-based techniques applied to conveyor belt condition monitoring. Following PRISMA guidelines, a search was conducted in the Scopus and Web of Science databases, and 80 studies were selected after applying predefined eligibility criteria. These studies were synthesized through quantitative bibliometric methods and structured qualitative thematic categorization. The findings reveal a significant increase in scientific output after 2020, as well as its geographic distribution and potentially the most influential contributions. The main research lines focus on damage detection, deviation detection, and foreign object detection. A clear transition is also observed from traditional image processing methods—such as filtering, segmentation, and geometric analysis—toward deep learning models, including YOLO, CenterNet, and hybrid architectures, with improvements in precision, speed, and stability. Nevertheless, challenges remain related to datasets representativeness, the heterogeneity of evaluation protocols, and variability in operational conditions. Finally, opportunities for advancement are identified through multimodal datasets, adaptive models, and lightweight solutions that facilitate integration into asset management systems and support scalable industrial adoption.

## 1. Introduction

In the mining industry, conveyor belts are a critical component of the value chain, as they ensure the continuous transport of large volumes of ore throughout the production process [1,2]. In modern applications, they can reach capacities of thousands of tons per hour and cover distances exceeding 100 km, typically operating at speeds between 1.5 m/s and 3 m/s continuously, 24 h a day [3,4]. From an economic perspective, the belt represents between 40% and 60% of the total system cost, making its reliability crucial to ensure operational continuity [5].

However, their exposure to heavy loads, impacts at transfer points, adverse environmental conditions, and prolonged operation make them particularly susceptible to failures such as belt wear, belt deviation, and material build-up [2,6,7]. These anomalies not only compromise operational continuity but also increase maintenance costs and pose safety risks when not detected promptly [6,8].

Hence, condition-based maintenance (CBM) is recognized as a widely adopted strategy in asset management, notably in mining, as it relies on monitoring the actual condition of equipment to determine both the need and timing of interventions [9,10]. A CBM program is structured around two complementary axes: diagnosis and prognosis. Diagnosis involves fault detection, localization of the affected component, and identification of the root cause. Prognosis, in contrast, aims to anticipate failure by estimating its probability, imminence, and the remaining useful life (RUL) [11,12].

Traditionally, the diagnosis of conveyor belts was conducted through manual inspections, a slow and imprecise process that relied heavily on the operator’s experience [13,14]. To overcome these limitations, classical computer vision was introduced, which combined image processing (e.g., adaptive thresholding, binarization, morphological operations, and Canny edge detection) with machine learning (ML) classifiers (e.g., SVM, AdaBoost, and k-NN) [15,16]. This integration enabled partial automation of visible damage detection; however, feature definition—such as textures, edges, or geometric patterns—still required manual design, and performance remained constrained by external factors such as dust, vibration, or poor illumination [15,17].

With the consolidation of smart industry technologies such as Artificial Intelligence (AI), the Internet of Things (IoT), and Big Data, computer vision has evolved toward increasingly sophisticated models based on deep learning (DL) [15,18]. Unlike the previous approach, CNNs and their variants (e.g., R-CNN, YOLO, SSD) are capable of autonomously learning hierarchical image representations, processing large volumes of information, recognizing patterns, and providing near real-time analysis [19,20].

Consequently, DL-based models have demonstrated improvements in precision, speed, and robustness, particularly in complex contexts such as underground mining, where noisy images, low contrast, and imbalanced datasets are common [19,21]. Even so, in certain scenarios, these advanced approaches do not always outperform traditional statistical methods, as multiple regression models can yield comparable predictive results. This suggests that model complexity alone is not a determining factor in performance [22].

In recent years, conveyor belt condition monitoring using computer vision-based techniques has attracted growing interest in both the academic community and the industrial sector. This trend is reflected in the sustained increase in publications reporting progress, both in the development of innovative solutions and their practical validation. Nevertheless, the heterogeneity of technical approaches and application scenarios makes it difficult to compare results and identify consistent progress in the field.

Although previous reviews on conveyor belt monitoring have been published, they have addressed the topic from more general perspectives, either focusing on the integration of artificial intelligence in transportation systems or on non-destructive diagnostic methods and remote sensing [23,24]. By contrast, the present review specifically focuses on computer vision-based techniques for conveyor belt condition monitoring.

The contribution of this study lies in providing a systematic and focused analysis that organizes the literature according to its main applications and synthesizes the methodological evolution from classical image processing to deep learning, enabling the identification of trends and future research directions. Table 1 presents the main differences compared to previous reviews.

To guide this review, the following research questions are proposed: What applications related to fault diagnosis have been addressed using computer vision? Which computer vision-based techniques have been employed? What results or performance metrics have been reported? What limitations and challenges have been identified?

**Figure 1:**
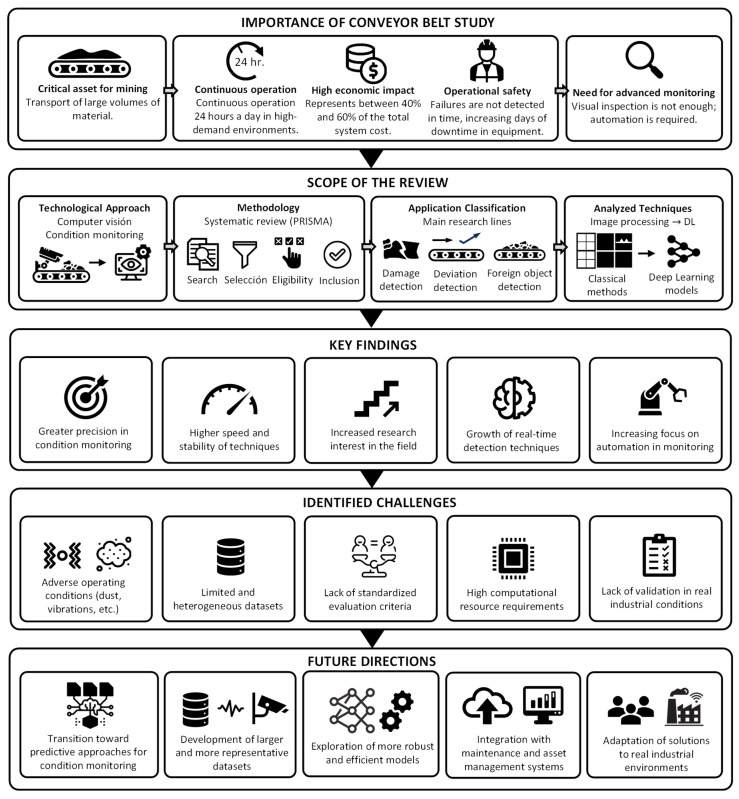
High-level diagram of the review, summarizing its scope, main findings, challenges, and future research directions in conveyor belt condition monitoring.

Figure 1 presents a high-level diagram of the review, summarizing its logical framework and overall organization. It highlights the significance of conveyor belt systems in the mining industry and outlines the main interconnected components, including the research focus, scope, and analytical dimensions in terms of condition monitoring.

The remainder of this article is organized as follows. Section 2 outlines the methodology. Section 3 presents the quantitative analysis of the included studies, focusing on bibliometric indicators and research trends. Section 4 presents the qualitative analysis, structured into thematic categories that enable a systematic comparison. Section 5 discusses the main findings, as well as the limitations, challenges, and future research directions. Finally, Section 6 presents the conclusions of the study.

## 2. Materials and Methods

The present systematic review was conducted in accordance with the PRISMA 2020 guidelines [25], given that this methodology is widely recognized for its rigorous and structured approach. It enables a comprehensive examination of the breadth and depth of the available literature, as well as the identification of knowledge gaps and thematic areas that require further investigation.

Regarding the eligibility criteria, records published between 2010 and 2025, written in English, and addressing computer vision-based techniques for conveyor belt condition monitoring were included. This time range was selected because, since the 2010s, research on artificial intelligence applied to industrial systems has increased significantly. Furthermore, since 2015, the development of deep learning models has substantially accelerated advances in computer vision for monitoring and automatic diagnosis applications [26,27]. Preprints, book chapters, review articles, and records not directly aligned with the research questions established in Section 1 were excluded.

The information search was performed using the Scopus and Web of Science (WoS) databases, selected for their broad coverage of peer-reviewed and indexed scientific journals, including high-impact publications in engineering and related technological fields. The search period extended from 11 September to 11 October 2025. No additional databases or sources were considered.

To guide the initial search, key terms such as “computer vision”, “maintenance”, and “conveyor belt” were combined using Boolean operators such as AND and OR. Table 2 presents the search queries used.

**Figure 2:**
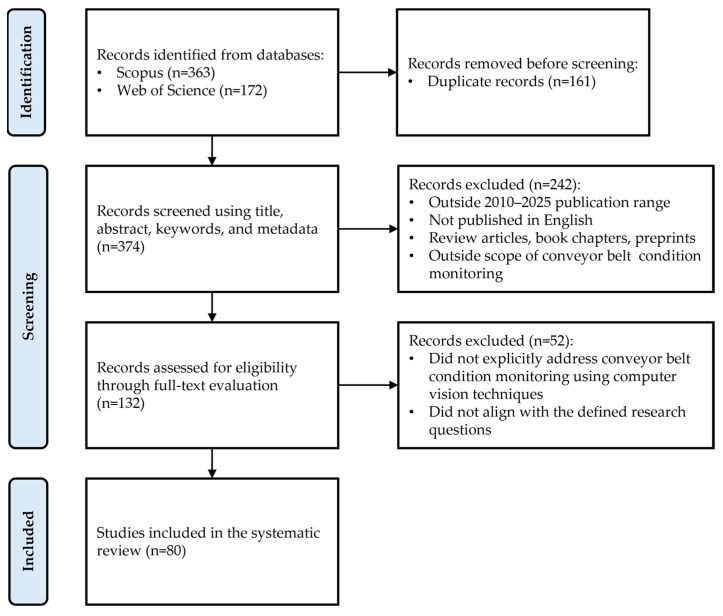
Study selection process.

After the identification of records and removal of duplicates, screening was conducted in two stages in accordance with the predefined eligibility criteria to exclude non-relevant records. In the first stage, titles, abstracts, keywords, and metadata were examined. In the second stage, full texts were assessed for eligibility. Both stages were performed independently by the four authors, and discrepancies were resolved by consensus. Figure 2 provides a detailed description of the study selection process.

Data collection was conducted using a double-entry matrix to record the information from each included study. On the one hand, bibliographic data were compiled, including publication year, authorship, institutional affiliations, citation counts, and keywords. On the other hand, relevant technical content was extracted, comprising the applications addressed, techniques employed, results obtained, and limitations described. The extracted information was cross-checked among the authors to ensure consistency.

Based on this information, a quantitative-qualitative approach was adopted. The quantitative analysis, of a bibliometric nature, examined the temporal evolution of publications, the productivity of authors and countries, as well as term co-occurrence. The corresponding results were represented through graphs generated using Python 3.14.3 and VOSviewer. The qualitative analysis consisted of a structured synthesis of the study contents, organized into thematic categories and presented in a comparative table.

No formal risk-of-bias tool was applied due to the heterogeneous and technological nature of the included studies, which primarily report engineering implementations with diverse performance metrics rather than standardized effect estimates. Instead, to enhance methodological rigor, a structured evaluation framework was defined to guide the qualitative analysis of the included studies, considering key aspects of study design and validation. The criteria adopted for this evaluation are presented in Table 3.

This systematic review was not prospectively registered in a review registry. No formal review protocol was prepared prior to conducting this study. The completed PRISMA 2020 checklist is provided as Supplementary Materials.

## 3. Quantitative Analysis of Included Studies

**Figure 3:**
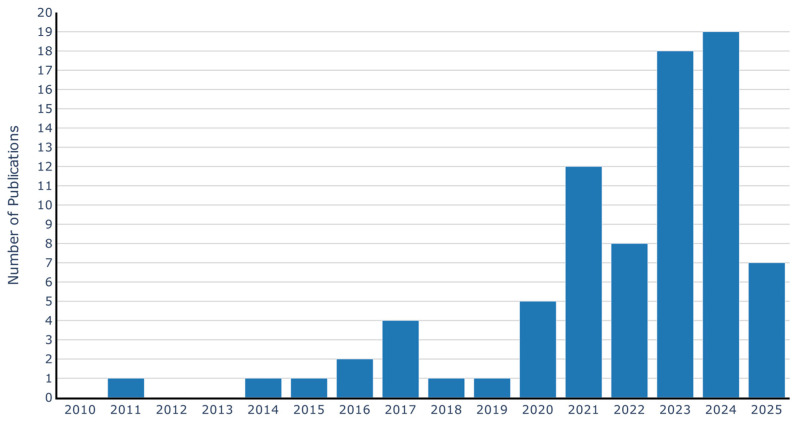
Number of publications per year.

This section presents the quantitative analysis of the 80 studies included. Figure 3 illustrates the number of publications between 2010 and 2025. Until 2019, the output remained low and relatively stable, ranging from one to three contributions per year. From 2020 onwards, a sustained increase is observed, reaching its peak in 2024 with 19 records. This trend demonstrates the growing interest of the academic community in condition monitoring of conveyor belts through computer vision-based techniques, thereby reinforcing the relevance of conducting the present review.

**Figure 4:**
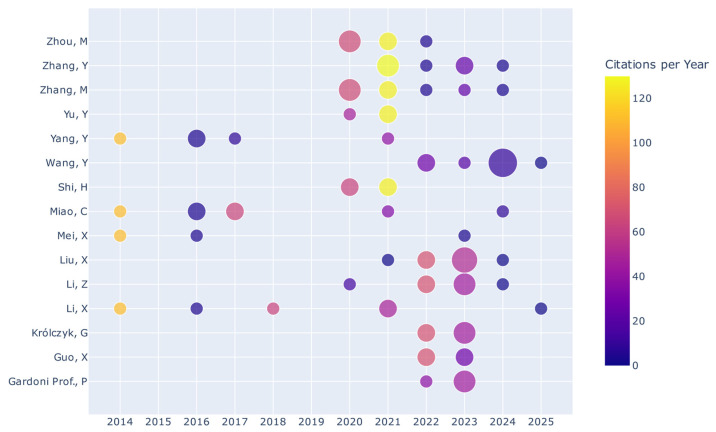
Author contributions and citations over time.

Figure 4 shows the annual contribution of the most influential authors during the period analyzed. In terms of productivity, Wang Y. exhibits the highest output, with nine publications, followed by Liu X. and Zhang M., who report eight each. Regarding academic impact, Li X. and Zhang M. produced the most cited works, both exceeding 225 citations. Furthermore, since 2020, there has been a notable expansion in collaborative networks, accompanied by the inclusion of new authors, which reflects the increasing diversification and dynamism of scientific output in this field.

**Figure 5:**
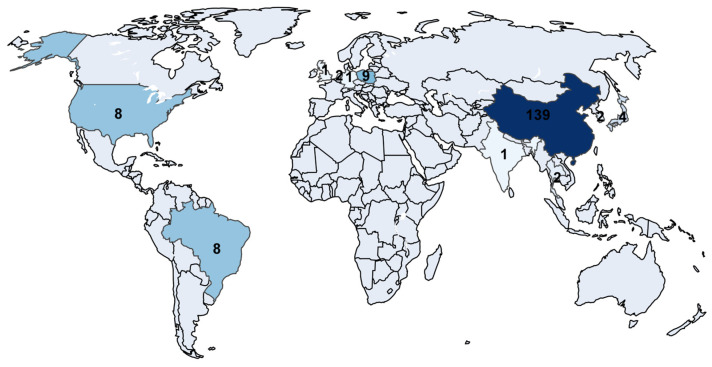
Geographical distribution of research contributions, where darker shades denote higher values and lighter shades denote lower values; numerical labels indicate the count per country.

Figure 5 presents the geographical distribution of scientific participation according to the institutional country of authors, reflecting the level of contribution by territory. China leads with 139 mentions, evidencing its central role in knowledge generation within the field, probably driven by its strong investment in research related to mining and mineral processing. To a lesser extent, Polonia, the United States, and Brazil make a noticeable contribution, while the remaining countries show only marginal participation.

**Figure 6:**
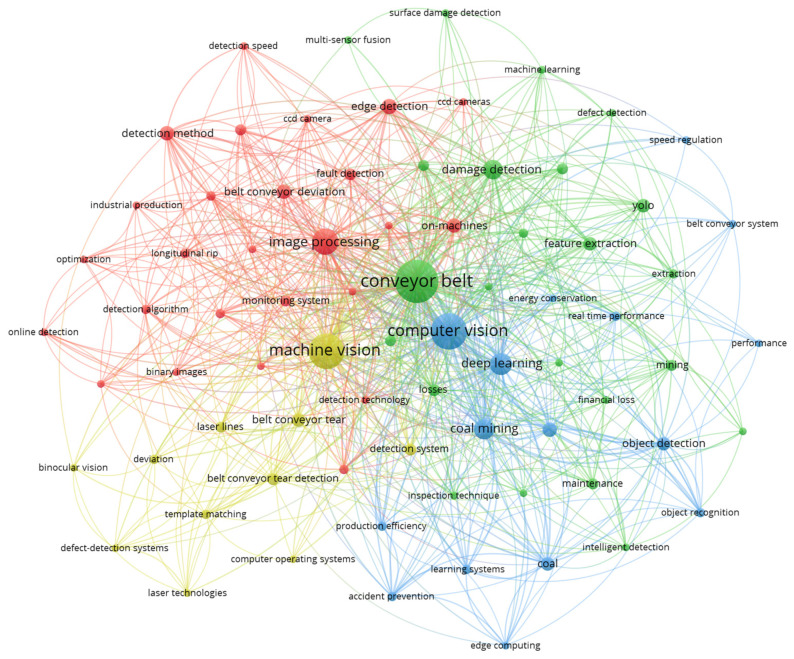
Keyword co-occurrence network on condition monitoring, conveyor belt and computer vision-based techniques.

Figure 6 describes a co-occurrence diagram that identifies the main thematic connections in the literature reviewed on conveyor belts. The visualization groups the related terms into four clusters. The red cluster is associated with image processing methods applied to monitoring and fault detection systems. The yellow cluster refers to machine vision and its applications in defect detection. The green cluster corresponds to the use of machine learning for intelligent damage detection. Finally, the blue cluster is linked to computer vision integrated with deep learning, aimed at optimizing mining and its associated strategic processes.

## 4. Qualitative Analysis by Thematic Categories

This section presents the qualitative analysis of the studies included in the review, grouped into four thematic categories: (a) belt damage detection, (b) belt deviation detection, (c) foreign object detection, and (d) other condition monitoring applications. This classification enables a comprehensive assessment of the methodological approaches, employed techniques, and obtained results in each category, with the aim of comparing and contrasting their contributions, identifying common patterns, and highlighting the main advances and current trends in intelligent conveyor belt monitoring.

### 4.1. Belt Damage Detection

The evolution of damage detection in conveyor belts has progressed from conventional visual inspection methods to intelligent systems capable of operating with stability in highly variable industrial environments. Over time, the need to ensure operational continuity, prevent critical failures, and maintain consistent diagnostics led to the adoption of advanced optical techniques, robust classification models, and later, DL approaches integrated with enhancement and stabilization mechanisms. The trajectory presented here indicates a sustained shift toward systems that are more autonomous, robust, and adaptable to the disturbances consistently present in contemporary industrial settings.

**Figure 7:**
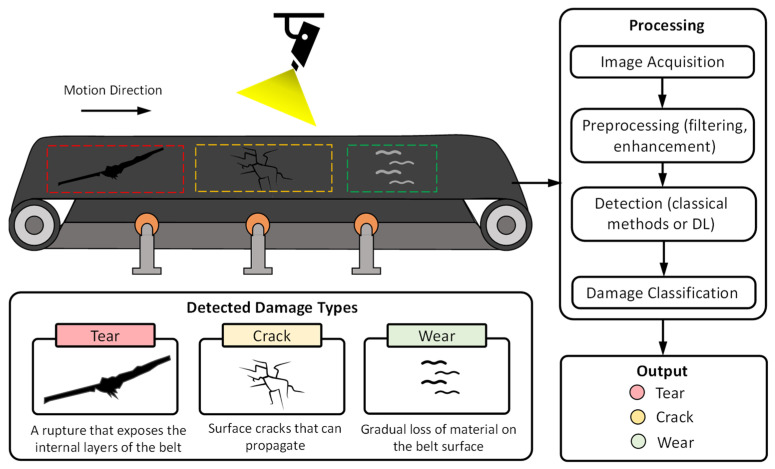
Conceptual framework for conveyor belt damage detection using computer vision, illustrating the processing pipeline from image acquisition to damage classification.

To provide greater clarity, Figure 7 presents the main components involved in conveyor belt damage detection using computer vision-based systems. The diagram synthesizes the overall processing pipeline, encompassing image acquisition, preprocessing, damage detection, and classification stages, as well as the types of defects commonly identified, such as tears, cracks, and wear.

In the earliest developments, the priority was to stabilize visual acquisition through controlled illumination, rapid segmentation, and contour extraction capable of isolating tears or other visible defects, thereby enabling basic diagnostics even with limited computational resources [21,28]. Building on this foundation, optimizing feature selection and dimensionality reduction became important to mitigate interferences produced by irregular textures or redundant correlations [22]. In a complementary manner, compression strategies were incorporated to ensure stable real-time transmission without sacrificing critical information, thereby supporting the feasibility of continuous monitoring [29].

As the need to operate under less controlled conditions increased, specialized filtering, optical stabilization, and geometric extraction assumed a more decisive role in sustaining damage detection under significant noise and environmental variation [30]. In this context, the combination of infrared and visible sensors proved particularly useful for compensating thermal fluctuations that altered surface contrast, thereby supporting a more consistent identification of longitudinal tears [31]. In parallel, 1D representations simplified profiles when 2D segmentation caused unnecessary delays [32]. Finally, the incorporation of laser-based measurement enabled a more precise delineation of damage contours and their longitudinal progression, reinforcing diagnostic accuracy [33].

It is worth noting that early approaches to conveyor belt damage detection remained strongly dependent on controlled acquisition conditions and manual feature engineering. Their performance was sensitive to parameter tuning and variations in surface appearance, limiting the consistent detection of tears, cracks, and related defects under changing operational conditions. This dependency constrained their applicability in heterogeneous industrial environments, where variability in transported material and surface condition produces irregular damage patterns.

**Figure 8:**
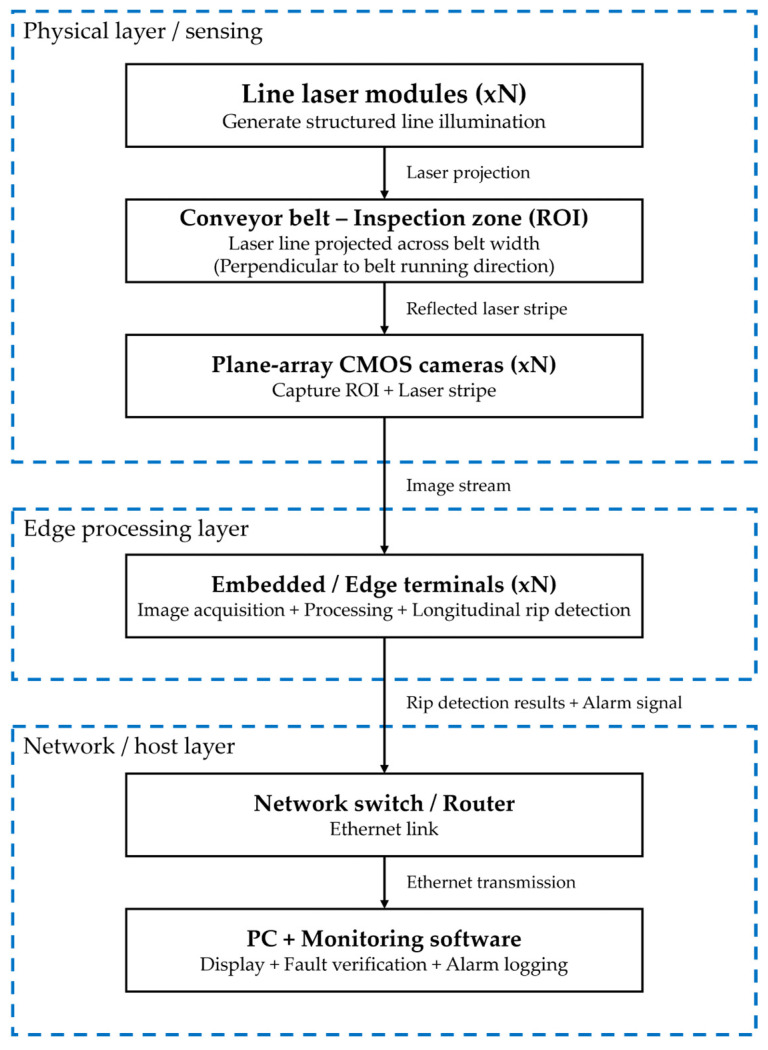
Conceptual block schematic of a traditional laser-based machine-vision system for online conveyor-belt longitudinal rip detection, based on the system description reported in Li et al. [33]. The projected laser stripe is captured by plane-array CMOS cameras and processed at the edge before transmission to a monitoring PC.

Figure 8 shows a simplified conceptual representation of a widely cited traditional laser-based machine-vision approach reported in the literature.

Starting in 2019, approaches integrating visual, acoustic, and geometric information became consolidated for the characterization of tears and cracks under complex operational conditions. The fusion of geometric patterns with templates and decision rules made it possible to distinguish prolonged ruptures even when the surface profile exhibited significant irregularities [34]. In addition, audio-visual integration provided an alternative means of complementing detection when the scene was affected by occlusions or insufficient illumination [35]. Subsequently, generative models expanded the representation of infrequent scenarios and reinforced multiclass identification in the presence of highly variable damage patterns [36]. The combination of images with point-cloud data also contributed to improving volumetric interpretation of deteriorated surfaces [37].

At this point, the incorporation of multimodal approaches also implied an increase in system complexity, particularly in terms of sensor synchronization, calibration requirements, and data processing. While this integration enabled more accurate detection of tears and complex defects, from an operational standpoint it tended to introduce additional constraints, as detection had to remain stable and continuous under conditions that do not always favor the coordination of multiple information sources.

Subsequently, the focus shifted toward more autonomous solutions capable of operating at the edge of the infrastructure and reducing reliance on centralized processing. This shift supported the development of lightweight architectures based on DNN designed to perform local inference without interrupting material transport [38]. In parallel, multiple-laser assistance improved the ability to detect microscopic tears in loaded belts, enhancing operational reliability [39]. This was complemented by 2D reconstruction and curvature analysis, which provided more detailed readings of early-stage imperfections [40]. Finally, the combination of edge-based processing and rapid segmentation proved essential in contexts where response time was a determining factor [41].

The availability of more extensive databases made it possible to refine deep architectures specialized for different types of damage, thereby strengthening accuracy in unstable scenarios. Within this framework, the integration of lightweight CNNs with efficient scaling promoted improved balances between processing speed and sensitivity to progressive wear [42]. Given the increase in the diversity of failure modes, it became necessary to incorporate deeper structures capable of maintaining stability even under inconsistent resolutions [43]. In parallel, methodological syntheses helped identify gaps related to class imbalance and the limited availability of real-world images. Conditional generative models reinforced this process by improving the discrimination of degraded patterns through the use of coherent synthetic data [7,20].

From a practical standpoint, the use of generative architectures such as MCC-CycleGAN introduces challenges beyond classification performance [7]. While they improve generalization in limited-data scenarios through synthetic data generation, they require computationally intensive training, leading to higher GPU demand, longer training times, and increased energy consumption. Therefore, their adoption should be evaluated not only in terms of accuracy, but also considering implementation cost, scalability, and return on investment (RoI).

Notably, the growing reliance on data-driven models also revealed constraints related to the availability and representativeness of training datasets. In many cases, the data used remained limited or insufficiently representative of the variability associated with the detection of tears and other defects, which tended to constrain generalization and affect performance when models were exposed to previously unseen scenarios.

**Figure 9:**
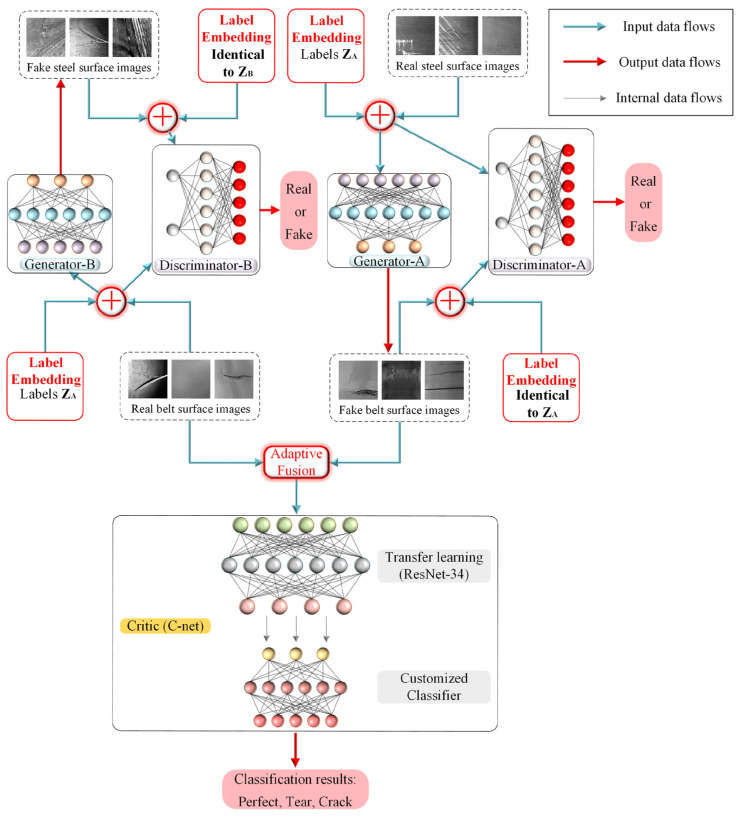
Topology of the proposed MCC-CycleGAN, integrating generators, discriminators and a transfer-learning-based Critic to translate images across domains, fuse synthetic and real data, and enhance damage classification in conveyor belts [7].

Figure 9 illustrates one of the most innovative computer vision-approaches of this period, combining DL and domain-transfer techniques to generate synthetic data and enhance damage classification in conveyor belts.

**Figure 10:**
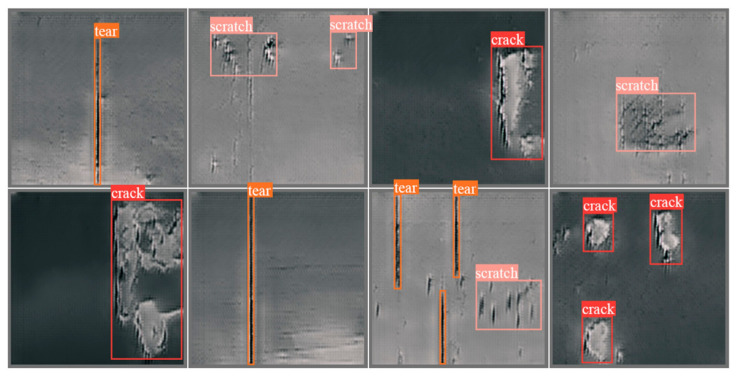
Visualization of different types of damage on conveyor belts obtained using DL-based algorithms, including representative models such as Faster R-CNN and YOLO [20].

Figure 10 illustrates how computer vision-based techniques, supported by DL detection algorithms, identify and visualize different forms of damage on the conveyor belt, such as tear, scratch, crack, and related patterns.

The interest in exploring complementary domains revealed that acoustic signals can anticipate tears when visual appearance is degraded by dust, vibration, or low illumination, enabling earlier diagnostics based on frequency-domain features [44]. In addition, the application of defogging techniques and Haar features contributed to stabilizing detection in environments with fog or irregular lighting [45]. In parallel, integrated segmentation-and-detection frameworks facilitated analysis of multiple forms of visible belt deterioration, including diffuse cracks or partially occluded damage [25]. Compressed-attention distillation also optimized the balance between processing speed and precision, supporting its incorporation into high-throughput industrial environments [26].

In tunnels and underground environments, detection was constrained by extreme illumination conditions and irregular spatial markers, which required models capable of locating cracks and estimating their progression in real time through additional calibrations [46]. To reinforce this process, image enhancement using invertible networks increased sharpness and stability in the presence of high-frequency noise [47]. In parallel, template-based methods and affine transformations accelerated structural comparison in areas with repetitive patterns [48]. Laser assistance with pyramidal configurations also increased robustness for analyzing extended longitudinal damage [49].

In logistics, the need for rapid inspection encouraged the use of shallow neural networks applied to filtering, segmentation, and contour extraction, which were suitable for detecting recurrent damage on homogeneous surfaces [50]. When geometry required correspondence between cameras, multiview configurations provided greater stability and reduced misinterpretations of deterioration-related patterns [51]. In 2024, adoption of optimized YOLO-based models enabled real-time estimation of the longitudinal extent of cracks and activation of automatic alerts according to severity [52]. Likewise, binocular vision with calibrated labels achieved more precise reconstruction of damaged regions, improving the 3D interpretation of surface deterioration [53].

In thermo-energetic environments, the combination of infrared radiation and optimized thresholding enabled damage identification even when visible contrast was insufficient, compensating for thermal disturbances [54]. The integration of multichannel attention and adaptive loss functions increased sensitivity to extensive or weakly defined tears [55]. Operationally, PLC-based systems and laser scanning preserved measurement stability by minimizing information loss from airborne dust [56]. In addition, YOLO implementations adapted to the operational context improved precision and processing speed in scenarios where damage morphology exhibited high variability [57].

In the most recent developments, multifusion platforms that combine X-ray, ultrasound, infrared, and vision modalities expanded detection toward non-visible internal faults and contributed to more comprehensive preventive diagnostic schemes [58]. In parallel, hybrid architectures integrating Retinex-based enhancement, efficient scaling, and lightweight attention mechanisms proved effective in maintaining stability in scenes with low brightness and deep noise in underground environments [59]. Improvements in optical damping and attention mixing further facilitated identification of cracks under intense vibration [60]. Deformable attention reinforced sensitivity to irregular surface-damage geometries [61]. Finally, use of optimized R-CNN combined with t-SNE-based feature reduction enabled more stable handling of dense and highly degraded textures [62].

Although a progressive technological consolidation can be observed in conveyor belt damage detection, a gap remains between experimentally reported performance and sustained operation under industrial conditions. In many cases, the proposed solutions were validated under controlled settings or over limited time horizons, which does not fully capture the variability or the actual evolution of tears, cracks, and other degradation mechanisms over time. This discrepancy underscores the need to advance toward more rigorous validation schemes that better reflect operational conditions.

Even so, the described panorama indicates a sustained progression toward systems capable of integrating complementary modalities, stabilizing degraded scenes, and recognizing complex patterns in real time. The convergence of deep vision, specialized sensors, and optimized algorithms has significantly reduced diagnostic uncertainty in environments characterized by dust, vibration, fog, heat, or structural interference. Although challenges related to standardization, generalization, and the availability of extensive datasets persist, the level of maturity achieved suggests the consolidation of fully autonomous solutions that are essential for extractive, energy, and industrial operations in which continuity and safety are critical.

Table 4, Table 5 and Table 6 present a summary of studies on traditional, hybrid, and deep learning-based approaches for conveyor belt damage detection, highlighting methods, obtained results, and identified limitations and challenges.

To complement this, Table 7 introduces a cross-category comparison based on accuracy, processing speed (FPS), and validation context (laboratory versus industrial), enabling a clearer identification of the key trade-offs between performance, computational cost, and real-world applicability.

### 4.2. Belt Deviation Detection

Lateral belt deviation constitutes a phenomenon distinct from structural damage because it does not always leave visible markers such as cuts or wear, complicating timely identification. Its progression depends on load distribution, tension imbalance, and roller geometry, generating positional patterns that may resemble ordinary oscillations. Although its immediate effects are less abrupt than tearing, persistent misalignment undermines transport stability, accelerates lateral deterioration, and increases the probability of mechanical interference if not corrected appropriately.

**Figure 11:**
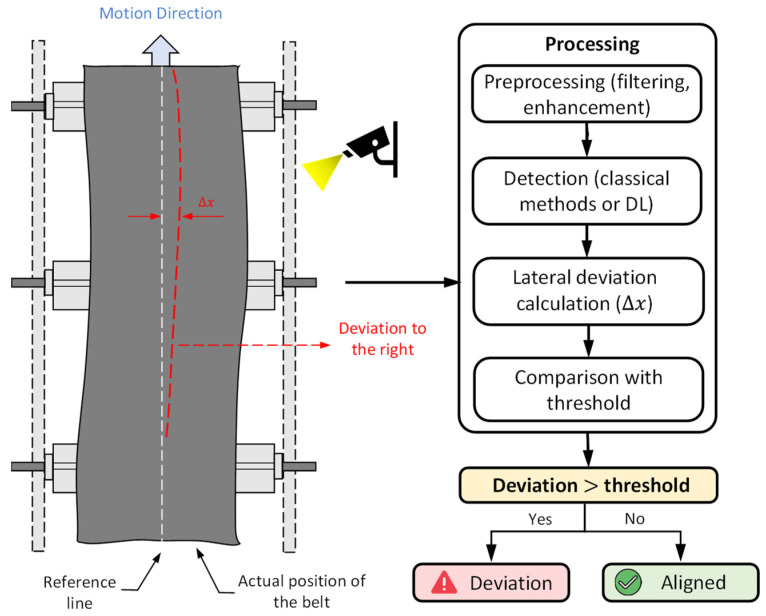
Conceptual framework for conveyor belt deviation detection, illustrating lateral displacement (Δx) and the processing pipeline for alignment assessment.

Figure 11 shows the key components involved in conveyor belt deviation detection. It illustrates the displacement (Δx) between the reference line and the actual belt position, along with the processing pipeline: preprocessing, detection (classical or deep learning–based), deviation calculation, and threshold comparison to determine whether the belt is aligned or deviated.

Early approaches concentrated on geometric approaches that reconstructed the belt axis through line-scan cameras, stabilized illumination, and binary segmentation, enabling displacement estimation with moderate computational requirements [21]. Building on these foundations, methods based on intensity variations were incorporated to compensate for uneven lighting, introducing indicators such as center displacement and edge torsion, which improved metric stability [63]. Complementarily, configurations focused exclusively on the geometric profile emerged, privileging simplicity and reduced sensitivity to noise during continuous operation [64,65].

It is worth noting that early geometric approaches to conveyor belt deviation detection remained strongly dependent on well-defined visual references and stable acquisition conditions. Since deviation does not always manifest through clear structural markers, these methods tended to struggle when edge visibility was compromised or when oscillatory patterns resembled normal belt motion, limiting their reliability in more complex operational scenarios.

**Figure 12:**
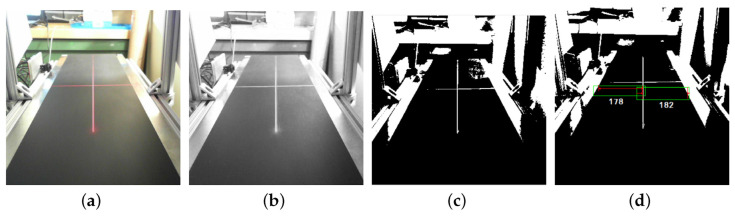
Processing sequence of a laser-based computer vision approach for estimating conveyor belt deviation: (**a**) original image with the laser line as a reference, (**b**) HSV conversion to enhance the laser line, (**c**) binarized image after threshold segmentation, (**d**) measurement of the distance between the belt edges and the laser line [63].

Figure 12 shows the sequence of image-processing steps used in a laser-based computer vision approach, from the initial capture to the extraction of the information employed to estimate the deviation.

With enhanced instrumental capabilities, inspection robots equipped with lightweight DL networks began to be integrated, expanding spatial coverage and correcting distortions through homography-based transformations [66]. Simultaneously, rapid filtering and edge-extraction techniques were optimized, demonstrating improved stability in the presence of dust and vibration [67]. This progression facilitated the incorporation of multi-source data for comprehensive monitoring and enabled the use of temporal models that characterized deviation in curved belts with greater operational coherence [68,69].

The incorporation of multisensor data and mobile inspection platforms also implied increased demands in terms of calibration consistency and spatial alignment. In deviation detection, where small positional variations can lead to misinterpretations, these factors tended to affect measurement stability, particularly over long trajectories or under variable loading conditions.

A relevant technical transition emerged with the adoption of adaptive models for continuous segmentation, which preserved edge coherence under varying illumination and provided stability in changing scenarios [70]. In parallel, HDR techniques combined with dual-baseline configurations offered a consistent alternative for underground environments with wide lighting variations [71]. Based on this progression, integrated architectures were developed to unify segmentation and detection within a single computational framework, broadening the operational scope of deviation estimation [25]. Additionally, stereoscopic systems with AprilTags strengthened triangulation processes [53].

Since 2024, attention has shifted toward deep architectures equipped with attention mechanisms, bidirectional fusion, and multiscale analysis, all of which reinforced axis delineation under optical noise, particularly in optimized YOLOv8 variants [72]. In parallel, hybrid methods combining enhanced Canny operators, guided filtering, and refined Hough transforms were systematized, achieving more stable contours under real operating conditions [73]. Inspection robots with RFID-based correction were also incorporated to reduce accumulated drift during long trajectories [74]. Finally, laser-intersection techniques supported more controlled geometric measurements [75].

**Figure 13:**
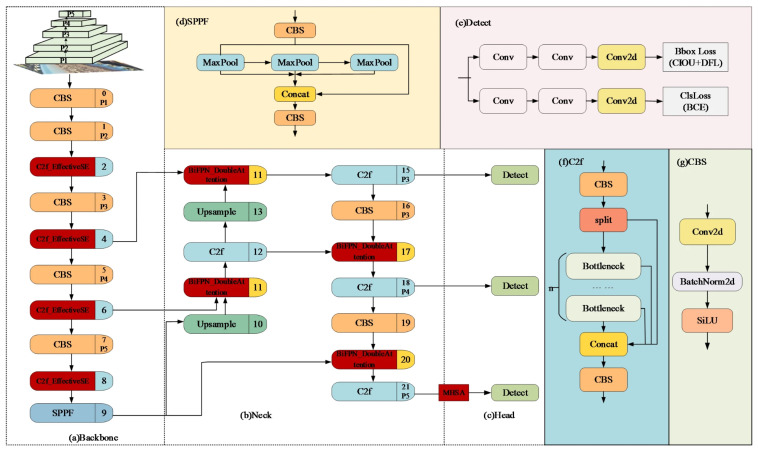
Improved YOLOv8 architecture: (**a**) backbone for initial feature extraction, (**b**) neck for multi-scale feature fusion, (**c**) head for feature refinement, (**d**) detection layer for object localization and classification of rollers and belt edges associated with deviation, (**e**) multi-scale pooling module, (**f**) feature fusion module, and (**g**) basic convolutional block [72].

Figure 13 illustrates a recent computer vision approach that incorporates targeted structural improvements to the YOLOv8 architecture for the real-time detection of conveyor belt deviation.

The growing use of deep architectures also introduced additional demands in terms of interpretability and robustness in deviation estimation. Unlike damage detection, where features are typically localized, deviation required maintaining consistent spatial referencing across the entire belt width. In this regard, models tended to exhibit sensitivity to slight variations in camera positioning or belt geometry, affecting the stability of inferred displacement patterns.

In the most recent period, 3D schemes based on binocular laser systems were developed to estimate deviation and flatness variations with short response times and suitable accuracy [76]. At the same time, ultralight networks such as FastBeltNet emerged, balancing temporal stability with low computational demand, which favors integration into industrial scenarios [77]. Multisensor platforms combining vision, infrared sensing, ultrasound, and X-ray imaging further strengthened robustness under demanding conditions [58]. In addition, IoT-based systems incorporated autonomous deviation monitoring within solutions oriented toward operational safety [78].

Although technological evolution improved detection capabilities, a gap remains between deviation estimation under controlled conditions and its tracking under continuous industrial operation. Sustained monitoring requires maintaining stable spatial references over long trajectories and under variable loads, aspects not always considered in experimental validation. This gap reinforces the need for more representative evaluation strategies that reflect the dynamic nature of deviation phenomena.

Overall, the technical progression reveals a transition from purely geometric and binary-segmentation approaches to systems incorporating deep vision, spatial reconstruction, and longitudinal correction mechanisms. Because deviation manifests through subtle and context-dependent displacements, research progressively adopted methods with enhanced sensitivity to edge continuity, spatial coherence, and temporal stability. Persistent challenges include environmental variability, the lack of standardized datasets, and the need to balance computational efficiency with metric consistency in long-distance monitoring. These aspects continue to shape the evolution of industrial surveillance strategies.

Table 8 and Table 9 present a summary of studies on traditional and deep learning-based approaches for conveyor belt deviation detection, comparing the techniques employed, the results obtained, and the identified limitations and challenges.

To complement this, Table 10 introduces a cross-category comparison based on accuracy, processing speed (FPS), and validation context (laboratory versus industrial), enabling a clearer identification of the key trade-offs between performance, computational cost, and real-world applicability.

### 4.3. Foreign Object Detection

In conveyor belt, object detection represents a distinct challenge compared with other failure modes, as it requires identifying external, unpredictable, and potentially hazardous elements whose appearance may be sudden and without precursor signals. While wear or misalignment evolve gradually and allow cumulative diagnostics, foreign objects demand algorithms with very short response times, stability under illumination variations, and consistent performance on fast-moving belts.

**Figure 14:**
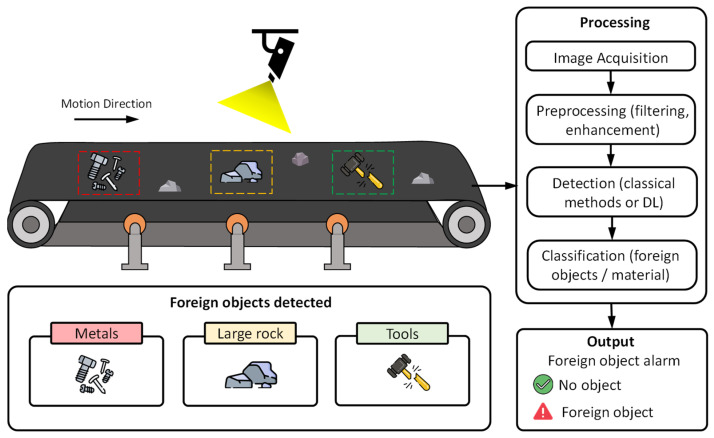
Conceptual representation of foreign object detection on conveyor belts using computer vision, highlighting typical hazardous objects (metals, large rocks, and tools).

Figure 14 presents the key components of foreign object detection on conveyor belts using computer vision, including potentially hazardous elements such as metals, large rocks, and tools, which are considered anomalies within the monitoring process.

Early approaches still relied on global image variations, such as histogram contrast, which made it possible to infer elements on the belt without explicitly distinguishing them [79]. As the field advanced, techniques emerged that interpreted local pixel changes as indicative signals, although their stability remained conditioned by illumination and noise [64]. Later, detection became more consistent through a YOLO model adjusted for foggy and blurred scenarios [80]. This was complemented by structured network compression, which reduced latency while preserving essential functionality [81].

It is worth noting that early approaches based on global variations or local changes exhibited inherent constraints for foreign object detection due to the absence of predefined structural patterns. Unlike other failure modes, the occurrence of foreign objects does not follow a progressive evolution, which made consistent identification difficult when visual conditions varied or when objects shared characteristics with the transported material, particularly under heterogeneous loading conditions.

Since 2023, efforts have focused on situations where objects were large or exhibited poorly defined edges. In this context, adaptive Retinex techniques, multiscale templates, and an optimized MLP were used to improve boundary delineation and contour clarity [82]. Variants of YOLO incorporated MSRCR, MSAM modules, and depthwise-separable convolutions to strengthen tolerance to redundant features [83]. Lightweight architectures based on YOLO combined with ShuffleNetV2 and SimAM were also proposed for edge devices, along with deep pruning processes aimed at reducing inference time [84,85].

Under these conditions, the increase in architectural complexity also implied higher demands in terms of response time and inference stability. In foreign object detection, where response must be nearly instantaneous, even small latencies can compromise system effectiveness, particularly on high-speed conveyor belts, where detection delays may reduce the window for timely intervention.

**Figure 15:**
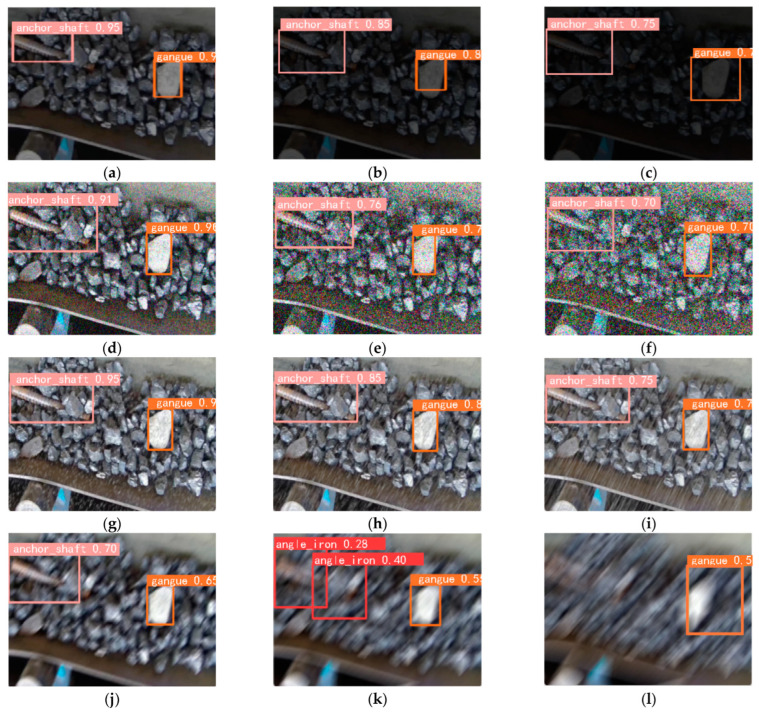
Detection of foreign objects using the improved YOLOv5s-PS model under adverse conditions: (**a**–**c**) low light, (**d**–**f**) visual noise, (**g**–**i**) mist or haze, (**j**–**l**) motion blur [85].

Figure 15 illustrates how computer vision-based techniques, supported by DL detection algorithms, enable the identification of foreign objects on conveyor belts even when the system operates under adverse conditions.

The adoption of embedded platforms encouraged the use of compact architectures, such as Nanodet with SIoU loss, which preserve speed and stability on ARM-Android processors [86]. Meanwhile, an improved YOLO model enhanced with IAT, CBAM, and a rotational head expanded the ability to represent elongated objects with variable orientation [87]. Additionally, an integrated framework combined segmentation and detection within a single structure, enabling the identification of foreign objects alongside other operational conditions [25]. Moreover, an anchor-free network with atrous convolution reinforced fine-detail extraction in metal mining environments [88].

Of course, the development of these solutions introduced additional trade-offs between computational efficiency and discriminative capability. While lightweight architectures facilitated their implementation in industrial environments, this balance tended to reduce sensitivity to small, deformed, or partially occluded objects, which typically require early and consistent detection.

**Figure 16:**
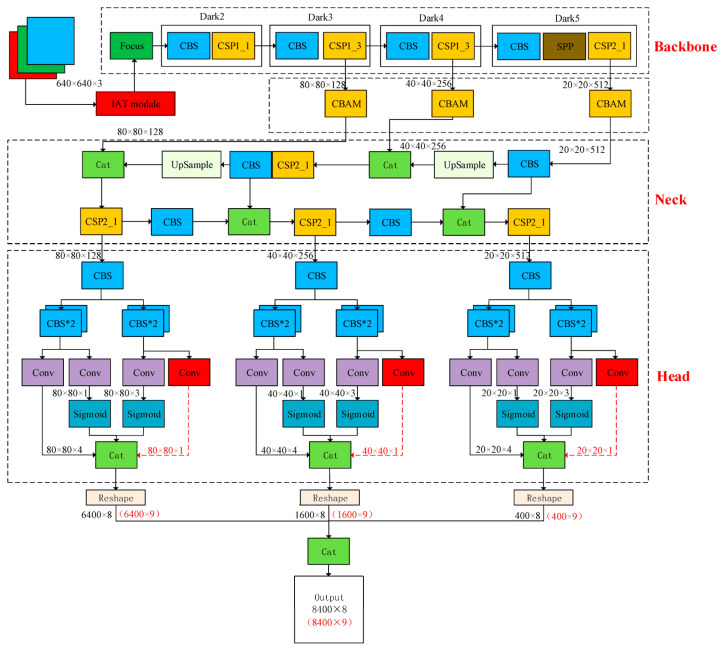
MO-YOLOX architecture that includes a CSP-based backbone with IAT and CBAM modules, a PAFPN neck for enhanced feature fusion, and a rotational head that generates angle-sensitive bounding boxes [87].

Figure 16 illustrates a recent computer vision approach that employs the improved MO-YOLOX architecture, integrating lightweight convolutions, attention modules, and angle-aware detection to optimize real-time foreign-object recognition on conveyor belts.

During 2024, stability in degraded images became a priority. In this setting, a YOLO model fine-tuned with attention mechanisms and specific activations preserved the semantic clarity of non-coal objects [89]. A separate line integrated a robotic manipulator triggered after detection, enabling the removal of objects before they reached sensitive process stages [90]. Another approach combined PLC, laser, and industrial cameras with YOLO to maintain detection in dusty environments [56]. When labeled data were scarce, GANomaly provided an identification criterion based on deviations from normal patterns [91]. Additionally, the incorporation of GhostConv into YOLO strengthened detection in manually annotated sets [92].

Recently, new approaches have emerged oriented toward early protection and hybrid frameworks. One strategy used YOLO to identify hazardous objects and trigger real-time alerts [93]. Another enhanced YOLO through attention mechanisms and EIOU, particularly useful in scenarios with limited data [94]. In industrial recycling contexts, the combination of line-scan cameras and segmentation enabled accurate discrimination of sharp objects [78]. Furthermore, multisensor systems integrated vision, infrared, ultrasound, and X-ray, delegating object detection exclusively to the camera [58]. In a different direction, the use of representations generated by foundation models enabled the segmentation of oversized material with minimal annotated data [95].

Within this evolving landscape, although technological developments have enabled advances in foreign object detection, a gap remains between identification under controlled conditions and performance under real industrial operation. The unpredictable nature of these objects, together with variability in shape, size, and sudden appearance, introduces conditions that are not always fully represented in experimental validation scenarios. This aspect suggests the need to consider evaluation strategies more closely aligned with the dynamic behavior of these events.

Thus, the historical trajectory reflects a progression from handcrafted-feature methods to deep architectures capable of operating under demanding conditions, incorporating multiscale attention, rotational mechanisms, and adaptive enhancement. Recent models balance accuracy, speed, and deployment on limited hardware, extending detection to large, deformed, or partially visible objects. The integration of segmentation, alerts, robotic manipulation, and multisensor fusion describes an increasingly cohesive operational ecosystem that supports the ongoing evolution of industrial monitoring strategies.

Table 11 presents a summary of studies focused on foreign object detection in conveyor belts, comparing the computer vision-based techniques employed, the results achieved, and the limitations and challenges identified.

To complement this, Table 12 introduces a cross-category comparison based on accuracy, processing speed (FPS), and validation context (laboratory versus industrial), enabling a clearer identification of the key trade-offs between performance, computational cost, and real-world applicability.

### 4.4. Other Condition Monitoring Applications

As complex equipment, conveyor systems are also exposed to failure modes derived from overheating, torsion, material accumulation, and other conditions that, although less frequently discussed in the literature, represent real risks for industries that must preserve asset reliability while maintaining sustainability criteria. This situation requires the supervision of parameters such as temperature and material flow, which are not typically visual. In such cases, information demands sensors and algorithms capable of interpreting thermal, geometric, or volumetric variations with sensitivity to operational dynamics.

Thermography, a consolidated technique within condition monitoring, was among the first to benefit from computer vision. Inspection using UAVs and infrared cameras enabled operators to overcome access restrictions and capture thermal anomalies in idlers, which were subsequently processed through signal-based techniques, although performance remained dependent on environmental conditions [96]. Later, the integration of local pixel-level differences enabled the identification of conditions such as empty-belt operation or human proximity, demonstrating the value of combining temperature and visual dynamics when the process exhibits rapid fluctuations [64].

**Figure 17:**
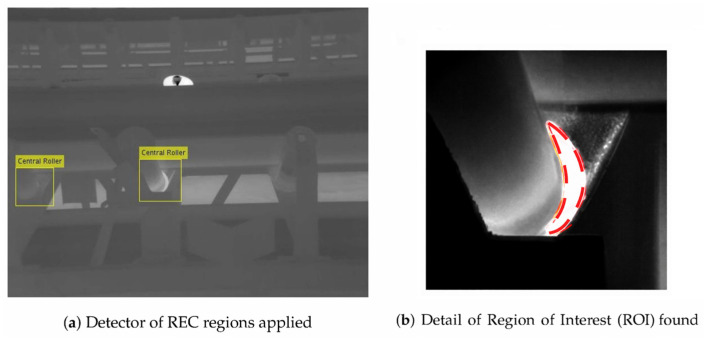
Thermal inspection visualization using computer vision: (**a**) automatic detection of central idlers using an ACF-based model, (**b**) ROI extraction and highlighting of thermally anomalous areas associated with potential faults [96].

Figure 17 shows how a classical computer-vision approach can identify anomalous regions on conveyor belt rollers using thermal information.

With emphasis on load accumulation, material-flow monitoring progressively focused on characterizing the transported volume and its influence on energy regulation. Initial solutions relied on background subtraction or particle analysis, which were useful but sensitive to contrast and granulometry [97]. Subsequently, the combination of cameras and laser devices provided more consistent volumetric measurements, although still affected by occlusions [98]. Deep learning techniques enabled the management of higher variability while avoiding the risks associated with radiological methods [99]. Finally, integrated models combined load, wear, and deviation within a single scheme, which increased data requirements [25].

In contrast, torsion in tubular belts introduces a strictly geometric problem associated with the longitudinal closure of the tube. The method based on regions of interest, edge operators, and line detection enabled the identification of the overlap with a moderate computational structure. However, its performance remained dependent on contrast and scene stability, factors that directly influence detection quality in environments affected by dust or irregular lighting [100].

Regarding failure modes associated with overheating, the problem became more complex with the inclusion of internal defects and ignition risks, which demanded broader multisensor systems. The integration of X-ray, infrared, ultrasound, and visual information enabled the detection of anomalous heat, internal wear, and deep cracks, with reduced false-alarm rates due to data fusion [58]. In parallel, eddy-current systems combined thermal surveillance with autonomous correction of misalignment and particle classification, provided that cleaning conditions and visual stability were adequate [78].

Given that idlers are essential components for overall belt performance, their monitoring has been revisited in recent years through approaches that have evolved toward deep networks aimed at improving localization and balancing thermal and structural features. Attention mechanisms and refined spatial-regression functions have increased prediction coherence in infrared images, facilitating detection in environments characterized by dust or thermal variation. Nevertheless, performance still depends on the availability of representative data and on the inherent variability of mining environments [101].

**Figure 18:**
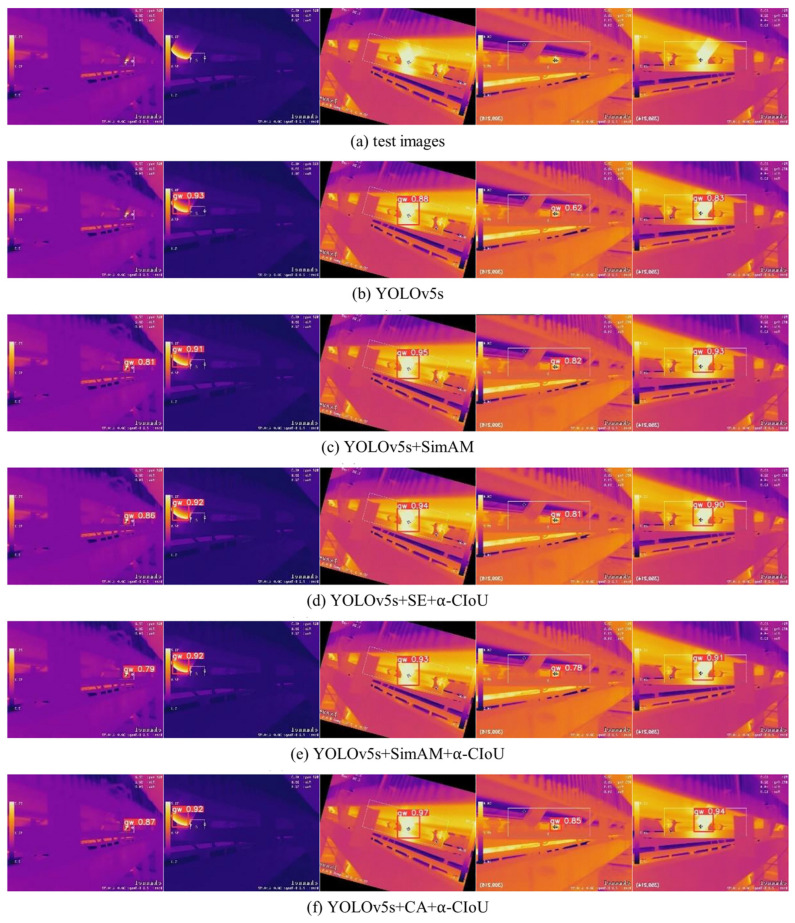
Comparison of DL models for detecting abnormal idlers using thermal imagery: (**a**) test images, (**b**) YOLOv5s, (**c**) YOLOv5s + SimAM, (**d**) YOLOv5s + SE + α-CIoU, (**e**) YOLOv5s + SimAM + α-CIoU, and (**f**) YOLOv5s + CA + α-CIoU [101].

Figure 18 shows how computer vision-based techniques, strengthened by DL-based detection algorithms, enable the reliable identification of abnormal idlers on conveyor belts using thermal imagery.

The diversity of phenomena addressed indicates that no method is universal or entirely transferable, since each scenario introduces specific constraints linked to failure type, material dynamics, and environmental conditions. This heterogeneity obliges industries and other stakeholders to select specific combinations of sensors, algorithms, and interpretative criteria that align with the nature of the problem. However, the solutions presented here reveal common lessons regarding modular integration, operational robustness, and the necessity of contextual validation. These insights support the design of tailored platforms aligned with the particular requirements of each conveyor system.

Table 13 presents a summary of studies focused on other condition monitoring applications in conveyor belts, comparing the computer vision-based techniques employed, the results achieved, and the limitations and challenges identified.

## 5. Discussion

This section discusses the main findings of the systematic review, providing an integrated interpretation of the results obtained from both the quantitative and qualitative analyses. In addition, it examines the limitations identified in the literature and outlines key challenges and future research directions for the development of computer vision-based conveyor belt monitoring systems.

### 5.1. Main Findings and Research Trends

The accumulated evidence indicates that computer vision-based monitoring of conveyor belt condition has concentrated primarily on three core problems: damage detection, deviation detection, and foreign object identification. This orientation reflects a clear research priority focused on failure modes that directly compromise operational continuity and safety, consistent with the principles of condition-based maintenance [9,11].

Early implementations relied on classical image processing techniques combined with traditional classifiers. These approaches enabled partial automation of visual inspection; however, they depended heavily on manually engineered features and environment-specific parameter tuning [33,34]. In practice, performance varied across sites and remained strongly influenced by the technical expertise required to define stable and relevant attributes [45,61].

With the consolidation of smart industry technologies, research progressively shifted toward DL-based models. Unlike earlier approaches, these systems learned hierarchical feature representations directly from data, reducing reliance on handcrafted descriptors [17,35]. This transition improved performance, but it also reshaped how detection tasks are conceptualized and how monitoring systems are structured for industrial deployment.

Over time, architectures such as YOLO and its variants became dominant solutions, particularly in applications requiring near real-time inference [55,57]. Their widespread adoption reflects industrial constraints in which latency and operational stability are critical. Rather than representing a purely methodological preference, this trend suggests a practical consolidation around architectures capable of balancing performance, computational efficiency, and deployment feasibility.

As this consolidation became more evident, research emphasis gradually shifted from structural innovation toward architectural refinement. Studies began incorporating attention modules, lightweight convolutional blocks, and optimization strategies tailored to limited-data scenarios [59,77,85]. The objective extended beyond maximizing detection accuracy alone, increasingly focusing on the trade-off between precision, computational cost, and deployability within resource-constrained environments [38,77,101].

Reinforcing the above, the bibliometric analysis allows the identification of patterns that provide a more grounded interpretation of the observed evolution. In particular, the concentration of contributions around vision-based and machine learning approaches, together with their transversal presence across the different categories, suggests that the field has tended to address heterogeneous problems through technologically convergent solutions. This behavior indicates that, beyond the differences between failure modes, detection strategies have been progressively unified around models capable of adapting to multiple operational conditions.

Likewise, the geographical distribution of research, largely concentrated in regions with strong mining activity such as China, is consistent with the emphasis observed on solutions oriented toward operational continuity. This correspondence suggests that the development of the field has not followed a purely technological logic, but has also been shaped by the need to operate in environments where system interruptions carry significant consequences, contributing to the prioritization of approaches that are robust, stable, and operationally viable.

### 5.2. Limitations

Even so, despite these advances, system-level robustness under real operating conditions remains a major challenge. Factors such as dust accumulation, uneven illumination, and mechanical vibrations continue to affect inference stability, even when advanced architectures are employed [45,47,80]. The recurrence of these constraints across independent studies suggests that reliable generalization across heterogeneous industrial environments is not yet fully achieved.

Against this backdrop, model generalization is further limited by data and training constraints. Domain shift becomes critical when training and deployment conditions differ, often leading to significant performance degradation in real-world scenarios. Likewise, overfitting persists in data-limited settings. Although techniques such as transfer learning and data augmentation partially alleviate this issue, models tend to capture dataset-specific patterns, reducing effectiveness under unseen conditions.

Compounding these challenges, heterogeneity in evaluation protocols hinders consistent comparison across studies. While metrics such as mean Average Precision (mAP), accuracy, and inference speed are widely reported, differences in datasets, environmental conditions, and validation procedures restrict objective benchmarking among models built upon similar architectural foundations [18,19].

From an implementation standpoint, additional constraints become evident. Many studies still rely on laboratory experiments or short-term evaluations that do not fully reflect sustained industrial conditions. Moreover, dependence on specialized hardware increases both system complexity and implementation costs, potentially limiting large-scale adoption.

Taken together, these limitations indicate that the effective deployment of such technologies extends beyond algorithmic performance alone. Rather, it requires a careful and well-balanced trade-off between accuracy, computational efficiency, maintenance demands, and hardware scalability. In this regard, although the field shows a clear convergence toward deep learning architectures, it remains in an operational maturation stage, where structural challenges continue to shape the transition from experimental prototypes to fully functional industrial systems.

With regard to the present review, it is important to acknowledge certain limitations inherent to its design and scope. The analysis was restricted to peer-reviewed publications written in English and indexed in selected databases, specifically Scopus and Web of Science. Although these sources provide broad coverage of scientific literature, relevant studies published in other languages, as well as records available in alternative repositories, patents, or non-academic sources such as industrial reports, technical standards, and proprietary implementations not formally disseminated, may have been excluded.

Another aspect to consider is that the possibility of publication bias or selective reporting cannot be entirely ruled out. The absence of negative or non-significant results in some studies may influence the overall perception of the reported performance and contribute to an overestimation of model effectiveness under controlled conditions. These considerations should be considered when interpreting the results of this review.

### 5.3. Future Perspectives

Building upon the limitations identified above, future research should adopt a structured and application-oriented roadmap to bridge the gap between experimental developments and industrial deployment. Several key directions emerge to guide the evolution of computer vision-based monitoring systems.

A first research direction involves the transition toward predictive models. Future approaches should move beyond reactive fault detection and advance toward prognostic systems capable of estimating the remaining useful life (RUL) of conveyor belts. This shift would enable earlier interventions and more effective maintenance planning under real operating conditions.

Another critical aspect concerns the development of realistic and representative datasets. Future datasets should incorporate operational variability, including changes in load, belt speed, environmental conditions, and material characteristics. In addition, the creation of multi-site datasets with standardized annotations and detailed metadata will be essential to improve model generalization and ensure reproducibility across studies.

Equally important is the design of robust and efficient models. Future research should focus on architectures capable of maintaining high accuracy while reducing computational complexity. This includes lightweight models suitable for edge deployment, as well as the incorporation of energy efficiency and hardware constraints into the evaluation process, thereby facilitating large-scale industrial adoption.

From an industrial perspective, the integration of monitoring systems with asset management platforms represents another key research direction. Detection outputs must be translated into actionable maintenance decisions, including prioritization, scheduling, and execution within CMMS environments aligned with condition-based maintenance strategies.

In parallel, the standardization of evaluation protocols remains a fundamental requirement. Future studies should adopt benchmarking frameworks that enable consistent comparison across different approaches. This includes multi-site validation, robustness analysis under environmental disturbances, and reproducible training, validation, and testing procedures.

Finally, the transfer of these technologies to real industrial environments must be prioritized. This involves addressing challenges related to sensor calibration, environmental variability such as dust, vibration, and illumination, as well as system maintenance and implementation costs. Moreover, evaluation frameworks should incorporate operational indicators such as equipment availability, downtime reduction, and resource efficiency to ensure the practical relevance of proposed solutions.

**Figure 19:**
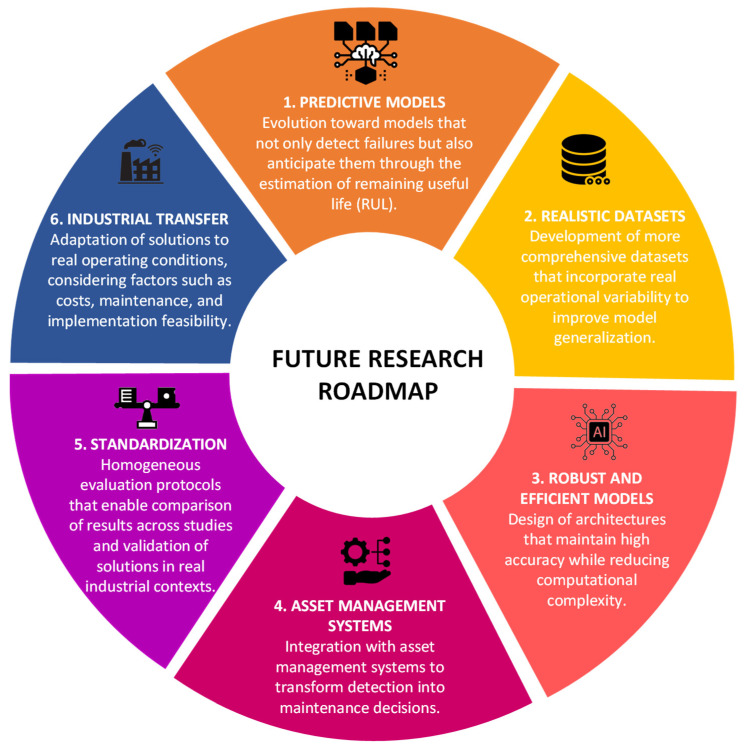
Future research directions in computer vision-based conveyor belt condition monitoring.

Figure 19 synthesizes the main future research directions derived from this review. These focus on addressing key challenges such as model robustness and efficiency, data representativeness, evaluation standardization, integration with asset management systems, and implementation in industrial environments.

## 6. Conclusions

This systematic review shows that research on computer vision-based monitoring of conveyor belt condition has focused primarily on damage detection, lateral deviation identification, and foreign object detection. These priorities reflect the operational demands of continuous transport systems, particularly in mining, where minimizing unplanned downtime and ensuring safe operation remain central objectives.

The literature reveals a clear technological transition from traditional image processing approaches toward consolidated deep learning architectures aimed at addressing environmental variability and enabling near real-time inference. This evolution is reflected not only in progressive improvements in accuracy and speed, but also in a structural transformation in how monitoring systems are conceived and deployed in industrial environments.

Nevertheless, significant challenges remain. Limitations related to dataset representativeness, cross-site generalization, computational constraints, and heterogeneity in evaluation protocols continue to hinder stable and scalable industrial adoption. Accordingly, the field can be characterized as being in an operational maturation stage rather than a phase of fully standardized deployment. Practical implementation must also account for computational complexity, edge-computing costs, sensor calibration requirements, and long-term maintenance demands in harsh environments.

Future progress should prioritize robustness under real operating conditions, standardized validation procedures, and the development of multimodal datasets validated in practice, rather than indiscriminate increases in architectural complexity. Moreover, deeper integration between vision-based detection systems and maintenance management platforms will be essential to translate automated detection into structured, data-driven decision-making.

A key next step for the field is the transition beyond fault detection toward prognostic frameworks in which visual indicators of belt degradation are linked to failure evolution models, maintenance thresholds, and RUL estimation. This integration will strengthen the contribution of computer vision not only to condition monitoring, but also to predictive maintenance and long-term asset-management decision-making.

## Figures and Tables

**Table 1:** Comparison between the present study and previous reviews.

Topic	This Study	Previous Reviews
Exclusive focus on computer vision	Yes	No
Organization by specific visual applications	Yes	Limited
Synthesis of evolution from classical image processing to deep learning	Yes	Partial
Comparison of techniques, results, and limitations within a unified thematic framework	Yes	Generic
Focus on implementation challenges in mining and industry	Yes	Less specific

**Table 2:** Initial search queries.

Database	Search Query
Scopus	(TITLE-ABS-KEY (“conveyor belt”) AND TITLE-ABS-KEY (“artificial vision” OR “computer vision” OR “system vision” OR “machine vision”) AND TITLE-ABS-KEY (“maintenance” OR “monitor*” OR “detect*” OR “predict*” OR “diagnos*” OR “prognos*”))
Web of Science	(TS (“conveyor belt”) AND TS (“artificial vision” OR “computer vision” OR “system vision” OR “machine vision”) AND TS (“maintenance” OR “monitor*” OR “detect*” OR “predict*” OR “diagnos*” OR “prognos*”))

**Table 3:** Criteria used for the structured qualitative assessment of included studies.

Criterion	Description
Validation scenario	Whether the method was validated in laboratory or real industrial conditions
Dataset availability	Whether the dataset is publicly available or privately collected
Dataset size	Whether dataset size is clearly reported to ensure reproducibility
Metrics completeness	Extent to which performance metrics are fully reported
Comparison baseline	Whether the study includes comparison with other methods or baselines
Real-time capability	Whether the method is suitable for real-time industrial implementation

**Table 4:** Synthesis of traditional computer vision studies for belt damage detection.

Ref.	Main Applications	Techniques Used	Results and Metrics	Limitations and Challenges
[28]	Automatic detection of tears in mining conveyor belts	CLAHE, median filtering, and hybrid segmentation (Otsu + mutual entropy) combined with geometric damage analysis	Achieved 90% accuracy for tears, 60% for scratches, and 45% for surface defects, with 0.4 s per image	Dependent on controlled lighting. Low robustness under dust or vibration in real-field environments
[21]	Online inspection of mining conveyor belts for longitudinal tear and deviation detection	Linear CCD camera with high-intensity line lighting, applying mathematical morphology, edge detection, and column-based segmentation	Effectively detected damage in laboratory tests, with 50 ms processing time and <10% CPU load	Requires precise lighting and calibration. Tested only in laboratory conditions
[29]	Compression and transmission of conveyor belts images for online damage detection	JPEG algorithm with 32 × 32 block linear difference and ABCD error control	CR = 20.26, MSE = 7.34. Achieved efficient compression and stable real-time transmission	Requires precise calibration of parameters and thresholds. Mistuning may cause artifacts or edge information loss
[30]	Detection of surface damage on conveyor belts	SICK Ranger C55 camera with 50 mW laser and Laplacian filters. Exposure set to 1300 µs	High image quality (2.46% null pixels) and accurate 3D damage detection	Requires precise calibration and stable environmental conditions. Sensitive to dust and vibrations
[31]	Detection of longitudinal tears in conveyor belts under mining environments	Binocular vision using CCD (visible) and infrared cameras. NSCT fusion, median filtering, and mathematical morphology	Achieved 96.3% accuracy and 18 ms processing time, enabled real-time detection	Requires frequent calibration and maintenance. Validated only in controlled laboratory conditions
[32]	Online detection of longitudinal tears in conveyor belts to improve safety and reduce failures	Conversion of grayscale images into 1D vectors, eigenfunction extraction, and adaptative threshold for diagnosis	High accuracy and noise robustness. Reliable real-time detection with optimal threshold of 53.98 and peak values 86.54–133.9 in experimental tests	Requires high-quality images and precise calibration. Not yet validated under industrial conditions with dust and vibration
[33]	Online detection of conveyor belt tears to reduce operational failures	CCD camera and laser line projection for acquisition. Sobel operator for edge detection. Gravity centers to identify tears	High accuracy and real-time speed with low localization error under simulated industrial conditions	Requires precise calibration and lighting control, sensitive to dust and vibration in industrial environments
[34]	Detection of longitudinal belt tears	Feature fusion with geometry, grayscale images, template matching, and DS evidence theory in a MATLAB GUI system	Accurately and stable detection of tear shape and extent with optimal threshold (μ_1_ = 139.7, μ_2_ = 100)	Requires auxiliary lighting and not yet fully real-time in industrial environments
[39]	Real-time detection of longitudinal tears in conveyor belts	Multi-line laser vision with median filtering, segmentation, and modified Sobel operator	Achieved 95.83% accuracy and 0.062 s per image, outperforming Otsu and fixed threshold	Needs clean optical setup and precise laser-camera calibration
[40]	Early detection of surface defects and wear on conveyor belts	Laser-based vision converting laser line to 1D signal and 2D reconstruction for defects	Achieved 92% accuracy and 94% recall, indicating efficiency and stable 2D laser reconstruction	Validated only in simulation. Depends on precise camera-laser calibration and lighting or dust conditions
[41]	Detection of surface tears on conveyor belts in coal mines	Industrial camera, smoothing filter, and Canny edge detection with sliding window to estimate damaged area	Average 0.4 s processing per image, meeting real-time industrial detection requirements	Needs stable lighting. Sensitive to dust and noise. Requires filtering and calibration optimization steps
[48]	Tear detection in conveyor belts using computer vision	Template matching and affine transform with laser line for keypoint detection	Achieved 96% accuracy and 26 ms per image, outperforming other methods	Relies on laser calibration and lighting. Affected by dust and vibration in real environments
[49]	Detection of longitudinal tears in conveyor belts using laser-assisted vision	Industrial camera with linear laser, pyramid processing, and template matching for anomaly detection	Reliable tear area identification with high visual effectiveness in simulated conditions	Needs accurate laser calibration and proper lighting. Lacks validation in real industrial settings
[51]	Detection of structural anomalies and belt wear	Multi-camera system with image processing and unique feature matching	High accuracy in anomaly detection and reduced human and material resources	Relies on camera sync and quality. Affected by lighting and fast motion
[53]	Visual monitoring of belt misalignment and breakage in production lines	Binocular vision with AprilTag markers for real-time displacement and depth measurement	Improved monitoring precision and stability, enhancing production efficiency	Needs camera calibration and lighting. Limited use in complex industrial environments
[54]	Visual detection of damage and tears in coal-fired power plant conveyor belts	Infrared CCD cameras, improved Otsu segmentation, FAST, and Hough transform	Over 98% accuracy, enabling effective monitoring in harsh industrial settings	Needs precise IR calibration and lighting; costly in dusty or vibrating environments

**Table 5:** Synthesis of hybrid computer vision studies for the detection of damage in belts.

Ref.	Main Applications	Techniques Used	Results and Metrics	Limitations and Challenges
[22]	Fault detection in conveyor belts (fractures, scratches, and tears)	EABC-SVM model optimizing SVM parameters and feature selection through an enhanced artificial bee colony algorithm	Achieved 95% accuracy, reduced features by 65%, and reached 100% detection for fracture and tears	Performance decreases under lighting and visual noise. It needs improved robustness in real industry environments
[35]	Detection of damage (tears and scratches) on conveyor belts using visual-acoustic fusion	CCD cameras and microphones; HOG, MFCC; PCA fusion; SVM, KNN, Random Forest models	Achieved 96.23% accuracy, 98.96% recall, detection 25.7 ms; outperforms vision or infrared	Sensitive to lighting and dust; needs deep models for higher robustness
[37]	Automatic surface damage detection in industrial belts	Camera–LiDAR fusion with Canny, DBSCAN, and RGB-depth data to reduce noise	Achieved 96.05% accuracy and 91.25% recall, outperforming image-only methods	Needs precise sensor calibration; affected by motion and lighting
[45]	Detection of longitudinal belt tears in foggy and unevenly lit environments	Haar features with modified dark channel dehazing and AdaBoost cascade classifiers	Achieved 98.4% accuracy, 96.9% recall, 99.9% precision, 0.1% FPR, 52.3 ms detection time	Relies on fog algorithm calibration; affected by dust or strong vibrations
[50]	Detection of breakage, cracking, and wear in conveyor belts	Machine vision filtering, segmentation, edge detection, and BP neural network classification	Accurate defect and location detection, improving logistics efficiency and safety	Dependent on image quality. Affected by defect variability and operating conditions.
[58]	Monitoring of conveyor belts	Multisensor fusion (vision, X-ray, IR, ultrasound, laser) using D-S evidence for prediction	False alarms reduced 50%→1% and missed faults 5%→2%, improving reliability	Needs accurate sensor calibration and high-quality data in real mining environments

**Table 6:** Synthesis of deep learning-based approaches for belt damage detection.

Ref.	Main Applications	Techniques Used	Results and Metrics	Limitations and Challenges
[36]	Crack and tear detection in conveyor belts	CDCGAN model with skip-layer and multiclass softmax for three damage types	Achieved 96.2% accuracy, outperforms DCGAN (88.3%) and CGAN (90.1%) in accuracy and convergence	Tested only in lab. Needs industrial validation
[38]	Detection of longitudinal rips on conveyor belt under real operational conditions	Real-time belt images processed by DNN-model on edge device for local inference	MobileNet-0.75 achieved 100% accuracy, 93% recall, and 96% F1-score, ensuring reliable real-time detection	Reliable systems are costly and complex, while simpler ones lack dependability
[42]	Rapid detection of conveyor belt damages to enhanced monitoring efficiency	MobileNetV2-YOLOv4 model with depth wise convolutions. Mosaic and CutMix data augmentation for robustness	Achieved 93.22% mAP and 70.26 FPS, surpassing original YOLOv4 in accuracy (+3.5%) and speed (+188%)	Possible feature loss from channel reduction. Lack of validation in real industrial environments
[43]	Intelligent detection of multiple damage types in mining conveyor belts	Enhanced YOLOv3 model with EfficientNet-B0 and transfer learning on 3000 images	Achieved 97.26% mAP and 42 FPS, improved accuracy (+10.4%) and speed (+45.9%)	Hardware-dependent and parameter-sensitive. Needs validation in real environments
[7]	Surface damage detection and classification on conveyor belts (tears, cracks, perfect state)	MCC-CycleGAN with generators, discriminators and critic; embedded labels; incremental fusion; hinge loss; transfer learning with ResNet-34	Achieved 0.969 mAP, 0.968 Macro-F1, 99.5% accuracy, with strong generalization on small datasets	Long training, heavy architecture, low early synthetic quality, and reliance on high-performance GPUs, which increases the cost of implementation and energy consumption
[20]	Detection of longitudinal tears and other damages in mining conveyor belts	Comparison of magnetic, X-ray, IR sensors and YOLOv5, Faster R-CNN, SSD models	YOLOv5m achieved 82.5% mAP and 128 FPS, balancing accuracy and speed best	Limited by lighting, noise, and data imbalance. Recommends transfer learning and GANs
[44]	Detection of longitudinal belt tears	MFCC feature extraction and enhanced DenseNet model applied to acoustic signals	Average accuracy of 95.42% for longitudinal tear detection	Sensitive to noise and microphone calibration. Needs further industrial validation
[46]	Real-time detection and classification of cracks in tunnel conveyor belts	YOLOv4 model optimized with TensorRT and CBCD dataset	Achieved mAP 0.85 for cracks, and 0.99 for digits, reaching 15 FPS in real time	Low accuracy for small cracks and lighting variation. Needs more data and frame fusion
[26]	Automatic detection of conveyor belt damages for mining safety	FKD-CenterNet model with knowledge distillation and channel-spatial attention	92.53% mAP, 93.8% recall, and 65.8 FPS, outperforming YOLOv3 and SSD	Sensitive to dust and low light. Needs improved robustness in real-field settings
[47]	Real-time conveyor belt monitoring in demanding underground coal mining	Improved INN with new res-block, GLOW splitting for HF noise removal, enhanced TGF, and multi-loss design	Excellent denoising. Outperforms popular methods. Meets industrial damage-detection requirements	Image quality heavily degraded by extremely harsh underground conditions
[51]	Detection of longitudinal tears in main mining conveyor belts with intelligent control	YOLOv5s model with industrial camera and edge computing module	Stable real-time detection triggers alarms and auto-stop, improving transport efficiency	Accuracy depends on speed sensor and image quality. High initial implementation cost
[25]	Intelligent monitoring of conveyor belts for load, deviation, objects, and damage	YOLOv5 + U-Net architecture combining detection and segmentation, trained on 4291 images with transfer learning	Achieved 97% detection and 100% segmentation accuracy at 90 FPS, outperforming YOLOv4 and Faster R-CNN	Lacks coverage of curved belts and harsh settings. Limited dataset reduces generalization
[55]	Real-time detection of longitudinal conveyor belt tearing	Improved YOLOv5s with MCA attention plus Alpha-IoU Loss and SIOU Loss	Higher detection accuracy and efficiency, with better handling of sample imbalance and long-tail distribution	Affected by harsh industrial conditions and persistent issues related to sample imbalance and long-tail distribution
[56]	Surface monitoring of conveyor belts for real-time damage and object detection	PLC S7-200 system with Hikvision cameras, 880 nm laser, and YOLOv7 model integrated with C# HMI	Detected 1 mm defects at 2–5 m/s with high accuracy and low false-positive rate	Needs calibration and long-term validation. Affected by dust, vibration, and temperature
[57]	Automatic detection of longitudinal belt tears	Linear CCD cameras and YOLOv7 model optimized with SimSPPFCSPC, EIoU, and SimAM	Achieved 94.6% accuracy and 110 FPS, fewer errors, validated in mine and lab tests	Complex setup and high data load. May misclassify linear debris as tears
[59]	Automatic detection of underground belt wear	Retinex-YOLOv8-EfficientNet-NAM model (RYAN) with low-light enhancement and attention module	Achieved 98.57% accuracy and 66 FPS, outperforming YOLOv8, YOLOv5, and Faster R-CNN	Needs validation in extreme mining and optimization for embedded hardware
[60]	Monitoring belt wear from cracks and degradation under vibration	Computer vision with light beam, spring damper, and YOLOv7-ACmix model	Achieved 88% accuracy, 93% recall, 83.7% mAP, 60 FPS, outperforming previous model	Needs calibration and field validation. Tested only in laboratory conditions
[61]	Detection of longitudinal and surface damages in conveyor belts under complex conditions	YOLOv8 model optimized with RepVit, MobileNet-V3-L, and deformable attention (DAT)	Achieved 93.7% mean accuracy and 25.8 FPS, enabling fast and reliable real-time detection	Affected by extreme variability. Needs fine-tuning for belt type and lighting conditions
[62]	Detection of surface damages in conveyor belts	Enhanced Faster R-CNN with MobileNet and t-SNE for dimensionality reduction	Higher accuracy in tears and breaks compared to VGG-19 and ResNet-18	Limited by damage pattern variability and lighting. Needs tuning for real-time use

**Table 7:** Analytical comparison of approaches for conveyor belt damage detection.

Approach Type	Subtype	Accuracy	Speed	Validation Context
Traditional	Classical vision	~90–98% (45–60 for complex defects)	~2–20 FPS	Mostly laboratory/controlled environments
	Laser/geometric	~92–96%	~16–55 FPS	Laboratory/simulated industrial
Hybrid Methods	ML + vision	~95–98.4%	~20–40 FPS	Laboratory + limited industrial validation
Deep Learning	General DL	~92–99.5%	~25–128 FPS	Mixed (laboratory + partial industrial)
	Lightweight DL (Edge AI)	~90–97%	~60–110 FPS	Emerging industrial deployment
	Advanced DL (GAN/multimodal)	Up to >99%	Not consistently reported	Mostly laboratory

**Table 8:** Synthesis of traditional approaches for belt deviation detection.

Ref.	Main Applications	Techniques Used	Results and Metrics	Limitations and Challenges
[21]	Online inspection of mining conveyor belts for longitudinal tear and deviation detection	Linear CCD camera with high-intensity line lighting, applying mathematical morphology, edge detection, and column-based segmentation	Effectively detected damage in laboratory tests, with 50 ms processing time and <10% CPU load	Requires precise lighting and calibration. Tested only in laboratory conditions
[63]	Fast detection of conveyor belt deviation	Linear camera and edge segmentation to measure deviation, variation and torsion, correcting noise and uneven lighting	Accuracy of ±15% in width and ±10° in torsion. High speed, precision and low noise sensitivity	Requires precise calibration. Affected by sudden lighting changes
[65]	Conveyor belt deviation detection for improved safety	Canny edge detection, Hough transform, and laser method implemented in LabVIEW	High precision and stability, 26 FPS, laser method more reliable with lower error	Tested only in lab. Needs periodic laser calibration and industrial validation
[67]	Real-time detection and correction of conveyor belt deviation.	Dark-channel + wavelet enhancement; ROI differencing; Canny; Hough lines; pixel-distance mapping	Deviation error ≤ 0.4 cm; response < 4.6 s. Clarity improved under dust	Dust, low light, noise, and camera stability limit robustness. Preprocessing essential
[70]	Online detection of belt deviation state	Adaptive segmentation and belt displacement quantification model	<5% relative error, 16.3 ms per frame, stable real-time detection under speed variations	Needs calibration and validation in industrial settings with lighting and vibration changes
[71]	Automatic detection of conveyor belt deviation in mining	Adaptive HDR image enhancement, Canny edge detection, and Hough transform	Achieved 99.45% accuracy and <1% error, stable operation under low light	Lower accuracy for small deviations; needs validation in real industrial environments
[53]	Visual monitoring of belt misalignment and breakage in production lines	Binocular vision with AprilTag markers for real-time displacement and depth measurement	Improved monitoring precision and stability, enhancing production efficiency	Needs camera calibration and lighting. Limited use in complex industrial environments
[73]	Intelligent detection of conveyor belt deviation state	Video preprocessing with ROI, noise reduction, enhanced Canny, Otsu, Hough transform, and least squares	Achieved 95.4% accuracy and 26 FPS, stable left-right deviation detection	Lower accuracy under low light or dust. Needs calibration and robustness improvement
[74]	Detection and localization of conveyor belt deviation	Robot with computer vision, Canny detection, Hough transform, and RFID sensors	Achieved 92.1% accuracy and 31 FPS, centimeter-level real-time localization	Needs vision-sensor synchronization. Affected by vibration and harsh mining conditions
[75]	Intelligent detection of conveyor belt deviation	Enhanced Canny with hybrid filter and Otsu, Hough transform, and Shi-Tomasi corners	Accurate detection under 50 ms per frame, meeting real-time monitoring needs	Needs laser calibration. Affected by dust and lighting variation in mining environments

**Table 9:** Synthesis of deep learning-based approaches for belt deviation detection.

Ref.	Main Applications	Techniques Used	Results and Metrics	Limitations and Challenges
[64]	Intelligent monitoring of conveyor belts to detect deviation, idling, foreign objects, and human presence	Computer vision using YOLOv3 model and local pixel difference	Accurate and real-time detection of multiple anomalies with high stability and automatic alerts	Requires threshold tuning and calibration under lighting and vibration changes. Mining-site validation is still required
[66]	Automatic detection of belt deviations using inspection robot	OM-SSD (optimized MobileNet-SSD), Hough transform, and geometric correction	RMSE 3.7 mm, MAE 4.4 mm, 135.5 ms, enabling real-time continuous monitoring	Needs precise calibration; sensitive to vibration, dust, lighting. Larger dataset recommended
[68]	Conveyor belt misalignment monitoring in complex manufacturing environments	Computer vision with industrial sensors, PLC, Zumlink radio, and IoT cloud integration	Error below 5.1% and stable transmission beyond 2.5 miles	Needs periodic calibration and long-term validation in real industrial settings
[69]	Evaluation and prediction of deviation state in curved conveyor belts	Hybrid ARIMA-LSTM model with computer vision for real-time detection and prediction	MAE 0.0617, 52.38 s training, RMSE reduced >20% vs. single models	Needs tuning and validation under industrial lighting and vibration variations
[25]	Intelligent monitoring of conveyor belts for load, deviation, objects, and damage	YOLOv5 + U-Net architecture combining detection and segmentation, trained on 4291 images with transfer learning	Achieved 97% detection and 100% segmentation accuracy at 90 FPS, outperforming YOLOv4 and Faster R-CNN	Lacks coverage of curved belts and harsh settings. Limited dataset reduces generalization
[72]	Automatic detection of lateral deviations in conveyor belts	Optimized YOLOv8 model with EffectiveSE, BiFPN_DoubleAttention, and MHSA, trained on 5800 images	Achieved 98.1% accuracy, mAP@0.5 99%, and 46 FPS, stable detection in complex environments	Needs precise calibration and large dataset. Sensitive to environmental variations
[76]	Three-dimensional detection of belt deviations and tears in mining conveyors	Binocular vision with structured laser and 3D point cloud analysis via segmentation-clustering	Achieved 97.37% accuracy and 20–30 ms processing, 7.5% improvement over initial detection	Sensitive to dust and smoke. Up to 6.5 cm error, needs optical calibration and maintenance
[77]	Real-time edge detection of conveyor belts for enhanced deviation correction	FastBeltNet dual-branch network with Ghost, Downsample, and Input Injection modules	mIoU 80.49%, 90.89 FPS, 8.23 GFLOPs, 895 k params, high industrial accuracy and efficiency	Needs validation under extreme lighting and vibration conditions still
[58]	Monitoring of conveyor belts	Multisensor fusion (vision, X-ray, IR, ultrasound, laser) using D-S evidence for prediction	False alarms reduced 50%→1% and missed faults 5%→2%, improving reliability	Needs accurate sensor calibration and high-quality data in real mining environments
[78]	Intelligent monitoring of conveyor belts in BECS using computer vision	MLX90640 thermal cameras, Raspberry Pi with OpenCV, stepper motors, YOLOv11n-seg, and IoT-Telegram alerts	97% thermal detection, 84.8% accuracy, 84.5% recall, <3 s response in real time	Needs precise calibration; sensitive to noise, dust, lighting; larger dataset recommended

**Table 10:** Analytical comparison of approaches for conveyor belt deviation detection.

Approach Type	Subtype	Accuracy	Speed	Validation Context
Traditional	Edge/geometric-based methods (Canny, Hough, seg-mentation)	~92–99.5%	~20–31 FPS	Mostly laboratory/controlled environments
	Vision-based + enhancement (HDR, filtering, ROI)	~95–99%	~20–30 FPS	Laboratory + limited industrial testing
Deep Learning	Detection models (YOLO, SSD, CNN-based)	~93–99%	~25–90 FPS	Mixed (lab + partial industrial validation)
	Advanced DL (LSTM, multimodal, IoT)	High (task-dependent)	Real-time (hardware-dependent)	Mixed/emerging industrial

**Table 11:** Synthesis of studies on foreign object detection.

Ref.	Main Applications	Techniques Used	Results and Metrics	Limitations and Challenges
[79]	Online analysis of ash content in conveyed clean coal	Machine vision with Canon 600D camera, gray histograms, and RBF, SVM, and MLR predictive models	LIB-SVM achieved R^2^ = 0.82 and RMSE = 0.16, stable and accurate real-time predictions	Needs clean images and good lighting. Accuracy affected by noise, dust, or vibration
[64]	Intelligent monitoring of conveyor belts to detect deviation, idling, foreign objects, and human presence	Computer vision using YOLOv3 model and local pixel difference	Accurate and real-time detection of multiple anomalies with high stability and automatic alerts	Requires threshold tuning and calibration under lighting and vibration changes. Mining-site validation is still required
[80]	Detection of foreign objects on coal mine conveyor belts	Improved YOLOv5 with CBAM, ASFF, and defogging-blurring preprocessing	Achieved 96.6% accuracy and 56.5 FPS, +4.8% over YOLOv5 baseline, outperforming SSD and YOLOv3	Dependent on video quality and lighting. Needs tuning and broader mining validation
[81]	Rapid detection of foreign objects in non-coal conveyor belts	YOLOv5 with hybrid compression, structured pruning, and distillation for edge devices	>95% compression, >90% reduction, +157.5% CPU, +70.3% GPU, accuracy > 88.2%	High compression causes underfitting and accuracy loss. Hardware and validation dependent
[82]	Detection of large foreign objects on coal conveyor lines	Machine vision with multi-scale Retinex enhancement, template matching, and MLP optimized by Gray Wolf	Achieved 98.8% accuracy in lab tests, >95% in Gaoyang mine field tests	Needs manual template design and calibration under lighting and vibration changes
[83]	Detection of large objects on mining conveyor belts	Improved YOLOv5 with Ghost, ECA, K-means++, and CIoU for optimized accuracy	mAP@0.5 95.8%, 42.3% fewer parameters, 38.6% FLOPs reduction vs. standard YOLOv5	Requires industrial validation. Affected by occlusion and extreme lighting variations
[84]	Foreign objects on coal mine conveyor belts	Improved YOLOv8n model with lightweight ShuffleNetV2 backbone and parameter-free SimAM attention	Achieved 95.6% accuracy, 1.6 M params, 4.7 GFLOPs, −48.4% params, −42% compute load	Performance drops with blur/noise. Needs better preprocessing and industrial testing
[85]	Detection of foreign objects on mining conveyor belts	YOLOv5-PS model with SimAM, C3-PConv, SIoU, and dark channel dehazing, trained on 17,483 images	Achieved 94.9% accuracy, 230 FPS, 56% lower inference time vs. YOLOv5s	Sensitive to lighting and image quality. Needs real-mine and embedded hardware validation
[86]	Real-time portable detection of foreign objects on coal mine conveyor belts	Enhanced Nanodet with SIoU, Shufflenet_V2, and Wiener filter implemented on ARM + Android	94.3% mAP@0.5, 27–33 ms (~30 FPS), outperforming YOLOv5n and standard Nanodet	Needs calibration and mining validation. Affected by noise, dust, and lighting variability
[87]	Detection of foreign objects on mining conveyor belts	Improved MO-YOLOX with IAT, CBAM, and rotational head trained on 8100 real images	Achieved 93.87% accuracy, mAP@0.5 93.68%, 25 ms/image, outperforming YOLOX-S, SSD, and S2A-Net	Not tested in real or embedded systems. Needs optimization for in-mine deployment
[25]	Intelligent monitoring of conveyor belts for load, deviation, objects, and damage	YOLOv5 + U-Net architecture combining detection and segmentation, trained on 4291 images with transfer learning	Achieved 97% detection and 100% segmentation accuracy at 90 FPS, outperforming YOLOv4 and Faster R-CNN	Lacks coverage of curved belts and harsh settings. Limited dataset reduces generalization
[88]	Detection of foreign objects on molybdenum conveyor belts	Anchor-free CenterNet model with atrous convolutions, Hourglass-104, and optimized sample balancing techniques	Achieved 90.9% mAP and 26 FPS, outperforming YOLOv3, SSD, and Faster R-CNN in accuracy and stability	Sensitive to lighting, dust, and belt speed. Needs more data and real-field optimization
[89]	Recognition of non-coal objects on mining conveyor belts	Optimized YOLOv5 with noise reduction, segmentation, and visual attention mechanisms	Achieved 88.14% accuracy for gangue, 87.10% for metals, 27 FPS real-time performance	Accuracy drops under uneven lighting and high speed. Needs environmental robustness
[90]	Automatic classification of minerals and foreign objects with intelligent robot	Improved YOLOv7 with CA and robot path optimization via APSO in MATLAB	mAP and F1 ≈90%, 50% faster operation than standard PSO	Needs fine-tuning of vision-robot integration and further mining environment testing
[56]	Surface monitoring of conveyor belts for real-time damage and object detection	PLC S7-200 system with Hikvision cameras, 880 nm laser, and YOLOv7 model integrated with C# HMI	Detected 1 mm defects at 2–5 m/s with high accuracy and low false-positive rate	Needs calibration and long-term validation. Affected by dust, vibration, and temperature
[91]	Intelligent detection of foreign objects on underground coal conveyor belts	Enhanced GANomaly with attention gate and U-shape generator for image reconstruction and anomaly detection	AUC 0.864 on 707 real images, further outperforming previous GAN models	Needs large defect-free datasets and has high computational cost
[92]	Automatic monitoring and detection of foreign bodies on coal mine conveyor belts	YOLOv8G model optimized with GhostConv convolution core for real-time detection	High accuracy and real-time performance under varied environmental conditions	Needs large labeled dataset. Performance depends on lighting and mining conditions
[93]	Recognition of foreign objects on coal conveyor belts for preventive protection	Computer vision with YOLOv5 and intrinsically safe cameras linked to edge computing	Accurate real-time detection and streaming, enhancing system stability and intelligence	Needs alarm calibration and mining validation. Relies on video and controller links
[94]	Automatic detection of foreign objects on conveyor belts	Enhanced YOLOv8 with attention mechanisms and EIOU optimization	Outperformed YOLOv8n with higher accuracy, mAP50, and mAP50–95 in real tests	Needs large labeled datasets and validation under varying industrial conditions
[58]	Monitoring of conveyor belts	Multisensor fusion (vision, X-ray, IR, ultrasound, laser) using D-S evidence for prediction	False alarms reduced 50%→1% and missed faults 5%→2%, improving reliability	Needs accurate sensor calibration and high-quality data in real mining environments
[78]	Intelligent monitoring of conveyor belts in BECS using computer vision	MLX90640 thermal cameras, Raspberry Pi with OpenCV, stepper motors, YOLOv11n-seg, and IoT-Telegram alerts	97% thermal detection, 84.8% accuracy, 84.5% recall, <3 s response in real time	Needs precise calibration; sensitive to noise, dust, lighting. Larger dataset recommended
[95]	Segmentation and detection of large minerals on conveyor belts	SAM-enhanced U-Net using SAM, ViT-B, and MAE with self- and semi-supervised learning	mAPₘ increased from 36.04% to 65.08% using 31 049 images, leveraging unlabeled data	Needs large datasets and high computation. Sensitive to mineral shape and size variability

**Table 12:** Analytical comparison of approaches for conveyor belt foreign object detection.

Approach Type	Subtype	Accuracy	Speed	Validation Context
Traditional	Classical machine vision and ML-based methods (histograms, SVM, MLP)	Moderate (R^2^ ≈ 0.82; lower robustness)	Real-time (not consistently reported)	Laboratory/controlled
	Vision with enhancement and feature engineering	~95–98.8%	Moderate (real-time feasible)	Lab + limited field validation
Deep Learning	Deep Learning models (YOLO, CNN-based)	~93–97%	~25–90 FPS	Mixed (lab + partial industrial validation)
	Optimized and edge-oriented DL models	~92–98% accuracy; RMSE ~3–5 mm	~30–230 FPS	Limited industrial/edge deployment
	Advanced and integrated systems (GAN, multisensor, IoT, robotics)	High but task-dependent (AUC, mAP improvements)	Rarely reported; highly dependent on architecture and hardware	Mostly laboratory/emerging industrial

**Table 13:** Synthesis of studies on other condition monitoring applications.

Ref.	Main Applications	Techniques Used	Results and Metrics	Limitations and Challenges
[96]	Thermographic inspection of conveyor idlers	UAV with thermal camera, signal processing, and field-cloud connected backend platform	Achieved ~85.7% precision, 100% recall, and ~92.3% F1-score using the MP + DT configuration at the 40 °C threshold	Depends on stable conditions. Needs sensor calibration and industrial validation
[64]	Intelligent monitoring of conveyor belts to detect deviation, idling, foreign objects, and human presence	Computer vision using YOLOv3 model and local pixel difference	Accurate and real-time detection of multiple anomalies with high stability and automatic alerts	Requires threshold tuning and calibration under lighting and vibration changes. Mining-site validation is still required
[97]	Material flow detection on conveyor belts to optimize energy and wear	Camera-microcontroller system with background subtraction, Canny, and particle analysis	All methods effective. Particle analysis showed best accuracy, reliability, and speed	Tested under controlled settings. Needs industrial validation with varied materials and lighting
[98]	Real-time load detection on conveyor belts for energy-efficient speed regulation	M1: Background subtraction, thresholding, morphological filtering. M2: Laser-line extraction, alpha-shapes contouring, and cross-sectional area calculation	Both methods M1 and M2 identified load levels. Laser method provided consistent cross-sectional measurements	M1: Lacks reliable height accuracy. M2: Requires complex processing and careful contour reconstruction
[99]	Coal quantity detection and classification for energy saving	Machine vision with camera-laser, preprocessing (skeleton, thinning, fusion) and Dense-VGG based on VGG16	94.34% classification accuracy, 0.270 s per image	Needs laser calibration. Affected by dust, vibration, and lighting in harsh mining conditions
[25]	Intelligent monitoring of conveyor belts for load, deviation, objects, and damage	YOLOv5 + U-Net architecture combining detection and segmentation, trained on 4291 images with transfer learning	Achieved 97% detection and 100% segmentation accuracy at 90 FPS, outperforming YOLOv4 and Faster R-CNN	Lacks coverage of curved belts and harsh settings. Limited dataset reduces generalization
[100]	Detection of overlap edge and twist in tubular conveyor belts	OpenCV processing using Prewitt, mathematical morphology, and Hough transform	<3-pixel edge detection error, showing high stability and reliability in industrial use	Needs precise calibration. Affected by vibration and lighting variation
[58]	Monitoring of conveyor belts	Multisensor fusion (vision, X-ray, IR, ultrasound, laser) using D-S evidence for prediction	False alarms reduced 50%→1% and missed faults 5%→2%, improving reliability	Needs accurate sensor calibration and high-quality data in real mining environments
[78]	Intelligent monitoring of conveyor belts in BECS using computer vision	MLX90640 thermal cameras, Raspberry Pi with OpenCV, stepper motors, YOLOv11n-seg, and IoT-Telegram alerts	97% thermal detection, 84.8% accuracy, 84.5% recall, <3 s response in real time	Needs precise calibration. Sensitive to noise, dust, lighting. Larger dataset recommended
[101]	Fault detection in conveyor belt rollers using infrared imaging	Improved YOLOv5 with coordinate attention (CA) and α-CIoU loss, trained on 4500 IR images	Achieved 93.7% accuracy, 95.3% recall, mAP@0.5 95.3%, 285 FPS, outperforming YOLOv7/v8	Limited dataset. Needs expansion and mobile device further optimization

## Data Availability

No new data were created or analyzed in this study. Data sharing is not applicable to this article.

## Supplementary Materials

The following supporting information can be downloaded at https://www.mdpi.com/article/10.3390/s26082527/s1: Table S1: PRISMA 2020 Checklist.

## Abbreviations

The following abbreviations are used in this manuscript:

AI	Artificial Intelligence
BiFPN	Bidirectional Feature Pyramid Network
CBM	Condition-Based Maintenance
CLAHE	Contrast Limited Adaptative Histogram Equalization
CMMS	Computerized Maintenance Management System
CNN	Convolutional Neural Networks
DBSCAN	Density-Based Spatial Clustering of Applications with Noise
DL	Deep Learning
DNN	Deep Neural Network
FPS	Frames Per Second
GAN	Generative Adversarial Network
HOG	Histogram of Oriented Gradients
HSV	Hue Saturation Value
IoT	Internet of Things
k-NN	k-Nearest Neighbors
MFCC	Mel Frequency Cepstral Coefficients
MAE	Mean Absolute Error
mAP	Mean Average Precision
ML	Machine Learning
PAFPN	Path Aggregation Feature Pyramid Network
PLC	Programmable Logic Controller
R-CNN	Region-Based Convolutional Neural Networks
RGB	Red Green Blue
RMSE	Root Mean Squared Error
ROI	Region of Interest
RoI	Return on investment
RUL	Remaining Useful Life
SSD	Single Shot Detector
SVM	Support Vector Machine
UAV	Unmanned Aerial Vehicle
WoS	Web of Science
YOLO	You Only Look Once
1D	One-Dimensional
2D	Two-Dimensional
3D	Three-Dimensional
